# A novel small molecule screening assay using normal human chondrocytes toward osteoarthritis drug discovery

**DOI:** 10.1371/journal.pone.0308647

**Published:** 2024-11-01

**Authors:** Philip R. Coryell, Paul B. Hardy, Susan Chubinskaya, Kenneth H. Pearce, Richard F. Loeser

**Affiliations:** 1 Thurston Arthritis Research Center, School of Medicine, University of North Carolina at Chapel Hill, Chapel Hill, North Carolina, United States of America; 2 Center for Integrative Chemical and Biological Drug Discovery, University of North Carolina at Chapel Hill, Chapel Hill, North Carolina, United States of America; 3 Rush Medical College, Chicago, Illinois, United States of America; Universite Paris-Saclay, FRANCE

## Abstract

Osteoarthritis (OA) is the most common form of arthritis and a leading cause of pain and disability in adults. A central feature is progressive cartilage degradation and matrix fragment formation driven by the excessive production of matrix metalloproteinases (MMPs), such as MMP-13, by articular chondrocytes. Inflammatory factors, including interleukin 6 (IL-6), are secreted into the joint by synovial fibroblasts, and can contribute to pain and inflammation. No therapeutic exists that addresses the underlying loss of joint tissue in OA. To address this, we developed and utilized a cell-based high-throughput OA drug discovery platform using normal human chondrocytes treated with a recombinant fragment of the matrix protein fibronectin (FN-f) as a catabolic stimulus relevant to OA pathogenesis and a readout using a fluorescent MMP-13 responsive probe. The goal was to test this screening platform by identifying compounds that inhibited FN-f-induced MMP-13 production and determine if these compounds also inhibited catabolic signaling in OA chondrocytes and synovial fibroblasts. Two pilot screens of 1344 small molecules revealed five “hits” that strongly inhibited FN-f induced MMP-13 production with low cytotoxicity. These included RO-3306 (CDK1 inhibitor (i)), staurosporine (PKCi), trametinib (MEK1 and MEK2i), GSK-626616 (DYRK3i), and edicotinib (CSF-1Ri). Secondary testing using immunoblots and cells derived from OA joint tissues confirmed the ability of selected compounds to inhibit chondrocyte MMP-13 production and FN-f stimulated IL-6 production by synovial fibroblasts. These findings support the use of this high throughput screening assay for discovery of disease-modifying osteoarthritis drugs.

## Introduction

Osteoarthritis (OA) is a disease of the articular joints characterized by the destruction of cartilage, synovial inflammation, and abnormal bone remodeling. It typically occurs in the knees, hips, hands, and spine, which can lead to loss of mobility and debilitating pain [1, 2]. Over 500 million individuals globally are affected by symptomatic OA, including 32 million in the U.S., which is expected to rise to 67 million by 2030 [3–5]. OA treatment costs the U.S. healthcare system over $27 billion annually [6] and even more in lost economic productivity due to OA disability. Accordingly, researchers in the pharmaceutical field have taken great interest in finding and testing novel therapeutics to both alleviate the symptoms of OA and slow its progression. Unfortunately, results to date have been disappointing, with many drugs demonstrating promising preclinical results but failing in clinical trials [7, 8]. Current OA management only targets symptoms and not the underlying disease-causing tissue destruction, resulting in unchecked disease progression and ultimately the need for joint replacement [5]. A therapeutic intervention that halts, delays, or reverses OA progression is a critical unmet need.

Because proteinases, including matrix metalloproteinases (MMPs) and aggrecanases, are important mediators of joint tissue loss in OA [9], attempts have been made to develop specific inhibitors of these enzymes. To date, these inhibitors have either failed in clinical trials or have produced unwanted adverse effects such as diffuse musculoskeletal pain [10]. To address this, we have developed a novel cell-based functional high-throughput screening (HTS) assay to detect non-cytotoxic compounds that inhibit MMP production rather than activity. This platform utilizes primary human chondrocytes treated with fibronectin fragments (FN-fs) that stimulate increased MMP-13 production [11] used as a readout. FN-fs are a relevant stimulus because they are found in OA cartilage and synovial fluid [12] and stimulate production of multiple catabolic and proinflammatory mediators by chondrocytes that mimic the OA chondrocyte phenotype [11, 13, 14]. MMP-13 was chosen as a readout because it is a type-II collagenase that is overexpressed and secreted by OA chondrocytes and is one of the primary drivers of cartilage loss in OA [15, 16]. This screening platform also includes testing the cytotoxicity of each compound by measuring intracellular esterase activity to ensure that inhibition of MMP-13 production is not caused by cell-death, but rather the result of inhibiting OA-relevant targets and pathways. Since OA is a multifaceted disease that affects multiple joint cell types, follow-up testing of compounds from the screen was performed in both OA chondrocytes and synovial fibroblasts.

Using this approach, we screened the small molecule Library of Pharmacologically Active Compounds (LOPAC^®^1280, Sigma), a commercially available compound library of 1280 annotated bioactive and pharmacologically relevant compounds that spans a broad range of biological pathways and has been utilized in other screening campaigns [17–19]. We also screened a curated compound library of kinase inhibitors in a dose response format. Kinases are druggable targets that have also been investigated in OA studies, and kinase inhibitors have shown encouraging results for mediating OA phenotypes in pre-clinical experiments. For example, lorecivivint, a dual DYRK1A/Cyclin-like kinase 1 inhibitor that blocks activation of the WNT pathway has been demonstrated to suppress chondrocyte MMP-13 production [20]. The objective of these experiments was to test the robustness of the HTS to discover compounds that reduce MMP-13 production in chondrocytes treated with FN-f and that could potentially lead to the development of disease modifying OA drugs (DMOADs). We incorporated lorecivivint as positive control for MMP-13 inhibition given its known inhibition of MMP-13 production by OA cells and because it has shown promise as a potential DMOAD [21].

## Materials and methods

### Antibodies

Primary antibodies used were anti-MMP-13 (Millipore Sigma, MAB3321) at 1:1000 dilution, anti-IL-6 (Millipore Signal, MABF41) at 1:1000 dilution, and anti-β-Tubulin (Cell Signaling, #2146) at 1:1000 dilution. Secondary antibodies used were anti-rabbit IgG, HRP-linked antibody (Cell Signaling, #7074) and anti-mouse IgG, HRP-linked antibody (Cell Signaling, #7076) at 1:2000 dilutions.

### Chondrocyte and synovial fibroblast isolation and culture

Normal chondrocytes were cultured from human ankle (tali) cartilage of 16 deceased tissue donors obtained through the Gift of Hope Organ and Tissue Donor Network (Itasca, IL). These tissues were collected from donors with no known history of arthritis and lack of morphological indications of OA as determined by the Collin’s score [22]. OA chondrocytes and synovial fibroblasts were isolated from tissue obtained from 10 OA patients who had undergone total knee arthroplasty at the University of North Carolina Hospital, Hillsboro, NC. Normal and OA tissues were collected between 7/28/20 and 8/23/23. The acquisition and use of de-identified human tissue from cadaveric donors and from surgical waste material was reviewed by the University of North Carolina Institutional Review Board and determined to not constitute human subjects research (IRB #14–0189).

OA tissue removed during knee arthroplasty and cadaveric tissue from normal donors were placed in containers with sterile saline and kept refrigerated or on ice until use which was within 48 hours (hrs). Tissue or isolated cells were not frozen at any time. Chondrocytes were isolated via enzymatic digestion of cartilage tissue and plated in high density monolayer cultures without passaging to maintain the chondrocyte phenotype using protocols as previously described [23]. Chondrocytes were cultured in medium consisting of DMEM/F12 supplemented with 10% fetal bovine serum (FBS) (VWR Seradigm; #97068–085). For experiments that required serum starvation, chondrocytes were washed twice with phosphate-buffered saline (PBS) and fed DMEM/F12 without serum for the times indicated in the experiment.

For synovial fibroblast isolation, synovium in sterile saline was minced using sterile scissors and a scalpel until all tissue was collected (approximately 20 minutes) and rinsed 3 times with cold PBS. Tissue pieces were placed in spinner flasks and incubated for 30 minutes in 1 mg/mL sterile filtered pronase in MEM alpha media containing 10% FBS. Contents of spinner flasks were transferred to a sterile 50 mL conical tube and centrifuged at 2000 rpm for 5 minutes at room temperature to pellet tissue and separate adipose. After centrifugation, adipose was removed and the remaining tissue was washed twice with PBS and incubated in a spinner flask with 1 mg/mL sterile filtered collagenase in 10% FBS MEM alpha media for 2-6hrs (depending on the size of the tissue sample). Cells were filtered through a 70-um cell strainer and centrifuged at 2000 rpm for 5 minutes at room temperature to pellet cells. Cell pellets were washed twice with PBS and resuspended in 10–20 mL (depending on the size of the cell pellet) of 10% FBS MEM alpha. Cells were counted and plated up to 20 million cells on 150 mm cell culture dishes. Synovial fibroblasts were passaged at confluency and used for experiments at passage 3. For each passage, synovial fibroblasts were plated at a density of 2–3 million cells per 150cm dish or about 20,000/cm^2^. For experiments that required serum starvation, synovial fibroblasts were washed twice with PBS and fed MEM alpha without serum for the times indicated in the experiment.

### Fluorogenic MMP-13 responsive probe

The fluorogenic MMP-13 activity probe consisted of peptide sequence Ac-C(Cyanine5)GPLGFRVK(BHQ3), which was designed based on previous MMP activity probes [24] and was synthesized by Cambridge Peptides (West Midlands, UK). For probe validation, human articular chondrocytes from donor ankles were cultured for three days and then washed with PBS and fed serum-free media and treated with either 0.1% DMSO or 1 μM lorecivivint as a positive control inhibitor. After 2hrs, cells were then treated with either 1 μM FN-f or PBS for 24hrs. The FN-f used was purified 42 kDa endotoxin-free recombinant FN7-10 (1 μM), prepared as previously described [25]. The FN-f consisted of domains 7–10 in native fibronectin, which contains the RGD cell-binding domain recognized by the α5β1 integrin [26].

Media was collected and then aliquoted into microcentrifuge tubes and inoculated with 0.5 mM APMA and 20 μM MMP-13 probe. Fluorescent readings (excitation 651 nm; emission 670 nm) were obtained every hour using a SpectraMax M2e microplate reader (Molecular Devices) and SoftMax Pro Software (Molecular Devices). Plates were stored at 37°C between time points.

### Compound sets

The library of pharmacologically active compounds LOPAC^®^1280 was purchased from Sigma-Aldrich Co. LLC (Cat. LO4200). Compounds are listed in S1 File. Alectinib (T1936), Brigatinib (T3621), Cerdulatinib (T2487), Crizotinib (T1661), Galunisertib (T2510), and SU14813 (T1661) were purchased from Chemspace (https://chem-space.com/). An additional 62 compounds were purchased from MedChemExpress (https://www.medchemexpress.com/) and are listed in S2 File.

### HTS screening protocol

All pipetting and aliquoting for HTS screening was performed via automated liquid handling system (Thermo Scientific Multidrop Combi Reagent Dispenser, #5840330). Normal human chondrocytes were first cultured in 150 cm tissue culture dishes in DMEM/F12 media with 10% FBS for three days to allow for recovery from enzymatic isolation. Cells were then washed 3 times with PBS and dissociated with 10 mM EDTA in PBS at 37°C for 20 minutes and plated on pre-stamped 384-well compound screening plates in phenol-red-free DMEM/F12 media supplemented with 0.1% FBS. Each screening plate contained wells reserved for controls. Next, cells were incubated at 37°C for 2hrs and then treated with FN-f (final concentration of 1 μM) in phenol-red-free DMEM/F12 and incubated at 37°C for 24hrs. Cells in control wells were treated with either FN-f and no compounds or PBS and no FN-f. After 24hrs, compounds were tested for cytotoxicity by staining cells with 2 μM calcein AM diluted in PBS for 20 minutes at 37°C, and then measuring fluorescence via a microplate reader (further details below). Next, to measure MMP-13 levels in the cell culture media, PBS containing 0.5 mM APMA and 20 μM MMP-13 probe was aliquoted into each well. Fluorescence readings (excitation 651 nm; emission 670 nm) were obtained via a microplate reader. Plates were stored in a 37°C incubator between time points.

### Immunoblotting

For immunoblots measuring secreted proteins, conditioned media was collected from cell culture plates and boiled for 5 minutes in 4x Laemmli sample buffer (Biorad, #1610747). For immunoblots measuring intracellular proteins, cells were washed with cold PBS, lysed with cell lysis buffer (Cell Signaling, #9803) containing phenylmethanesulfonyl fluoride (Sigma-Aldrich, #93482) and Halt^™^ Phosphatase Inhibitor Cocktail (Thermo Scientific, #78420) and boiled for 5 minutes in 4x Laemmli sample buffer. Protein samples were run on an electrophoresis gel (Biorad), transferred to a nitrocellulose membrane (Biorad), blocked for 1hr. with 5% non-fat milk in Tris-buffered saline with Tween 20 (TBST) at room temperature, then incubated at 4°C overnight with primary antibody diluted in 5% non-fat milk in TBST. Membranes were washed 3x with TBST and stained with HRP-linked secondary antibodies diluted in 5% non-fat milk in TBST for 1hr. at room temperature. Membranes were washed 3x with TBST and HRP-linked antibodies were detected using ECL substrate (Biorad, 1705060S) and an imager (Azure Biosystems, model c600).

### Cell viability assays

Cell viability assays were performed with calcein AM and ethidium bromide homodimer staining. Reagents and protocols were from the Thermo Scientific LIVE/DEAD Viability/Cytotoxicity kit for mammalian cells (#L3224). The number of live and dead cells was determined by counting the number of calcein AM and ethidium homodimer-1-stained cells, respectively, from fluorescence microscopy images obtained using an EVOS M5000 microscope. Cell segmenting and counting was performed using CellProfiler (https://cellprofiler.org/). For Figs 3, 5 and 6, the percent of live cells from cell viability assays was calculated using the formula:

$$\%Live=100*\frac{live\;cells}{live\;cells+dead\;cells}$$

For high-throughput screens, only calcein AM staining was used to prevent interference between ethidium homodimer-1 and the MMP-13 fluorogenic probe. Accordingly, in Table 1 and Fig 4, the percentage of live cells was measured via calcein AM staining 24hrs after compound treatment and compared to a live control (PBS treated cells; 100% live) and a dead control (cells treated with 70% MeOH; 0% live).

**Table 1:** 20 LOPAC compounds with the greatest inhibition of MMP-13 production.

Name	%Inhib	%Live	Description	Class	Action
Suramin sodium salt	100	93	A reversible and competitive inhibitor of protein tyrosine phosphatases 1	P2 Receptor	Antagonist
Gossypol	100	64	Natural product from cotton seeds with a variety of cell biological activities. Proapoptotic, antimalarial, PKC inhibition	Apoptosis	Inducer
Mitoxantrone	100	58	DNA synthesis inhibitor	DNA Metabolism	Inhibitor
Thapsigargin	100	31	Potent, cell-permeable, IP3-independent intracellular calcium releaser	Intracellular Calcium	Releaser
BBMP	100	20	Mitochondrial permeability transition pore (PTP) inhibitor	Cell Stress	Inhibitor
Bay 11–7085	100	19	Inhibits cytokine induced IkB (Inhibitor of NFkB) phosphorylation	Cell Cycle	Inhibitor
Bay 11–7082	100	14	Inhibitor of cytokine-induced IKB-alpha phosphorylation	Phosphorylation	Inhibitor
Calcimycin	98	96	Ca2+ ionophore used to potentiate responses to NMDA, but not quisqualate glutamate receptors	Intracellular Calcium	Unspecified
Emetine dihydrochloride hydrate	94	84	Apoptosis inducer; RNA-Protein translation inhibitor	Apoptosis	Activator
NF 023	91	100	Potent, selective P2X1 receptor antagonist	P2 Receptor	Antagonist
Stattic	90	23	Irreversible STAT3 activation inhibitor	Gene Regulation	Inhibitor
Ouabain	86	77	Blocks movement of the H5 and H6 transmembrane domains of Na+-K+ ATPases	Ion Pump	Inhibitor
Cantharidic Acid	86	81	Protein phosphatase 1 (PP1) and 2A (PP2A) inhibitor	Phosphorylation	Inhibitor
1-(4-Hydroxybenzyl)imidazole-2-thiol	85	100	Dopamine beta-hydroxylase inhibitor	Dopamine	Inhibitor
CGP-74514A hydrochloride	83	90	Cdk1 inhibitor	Phosphorylation	Inhibitor
Kenpaullone	81	86	Potent inhibitor of CDK1/cyclin B, CDK2/cyclin A, CDK2/cyclin E, and CDK5/p25	Phosphorylation	Inhibitor
Reactive Blue 2	81	98	P2Y receptor antagonist; most potent antagonist for ATP-activated channels	P2 Receptor	Antagonist
MK-886	81	66	Potent and specific inhibitor of leukotriene biosynthesis	Leukotriene	Inhibitor
MK-933	81	85	Positive allosteric modulator of alpha7 neuronal nicotinic acetylcholine receptor	Cholinergic	Modulator
TBBz	80	100	Cell-permeable casein kinase 2 (CK2) inhibitor	Phosphorylation	Inhibitor

“%Inhib.” is the percentage of MMP-13 inhibited as described in the Methods. “%Live” is the percentage of live cells as described in the Methods. Compound name, description, class, and action were provided by Sigma.

### Data and statistical analysis

Microsoft Excel and GraphPad Prism were used for all data analysis, statistical calculations, and graphing. To calculate % MMP-13 inhibition in the HTS assays, the MMP-13 probe fluorescence detected at 24hrs. in the unstimulated chondrocyte control wells without inhibitors was set as the basal level of MMP-13 production. The MMP-13 probe fluorescence detected 24hrs after FN-f treatment in chondrocyte wells without inhibitors was set as the maximal MMP-13 production. Percentage of MMP-13 inhibited was calculated using the formula:

$$\%\;inhibition=100*\left(1-\frac{\text{x}-\mu_{u}}{\mu_{\text{s}}–\mu_{\text{u}}}\right)$$

Where x = sample fluorescence, μ_u_ = mean unstimulated control fluorescence, and μ_s_ = mean FN-f stimulated control fluorescence. Normal distribution was determined by Shapiro-Wilk test before Z-prime factor testing for assay robustness. Protein quantifications from immunoblots were obtained using the Fiji image processing package for ImageJ. Kolmogorov-Smirnov or Shapiro-Wilk test (when n<5) was used to determined normal distribution. Statistical significance was calculated using one-way ANOVA and Dunnett’s multiple comparison correction. Adjusted p-values were considered significant when < 0.05.

## Results

### Screen design and MMP-13 probe validation

**Fig 1:**
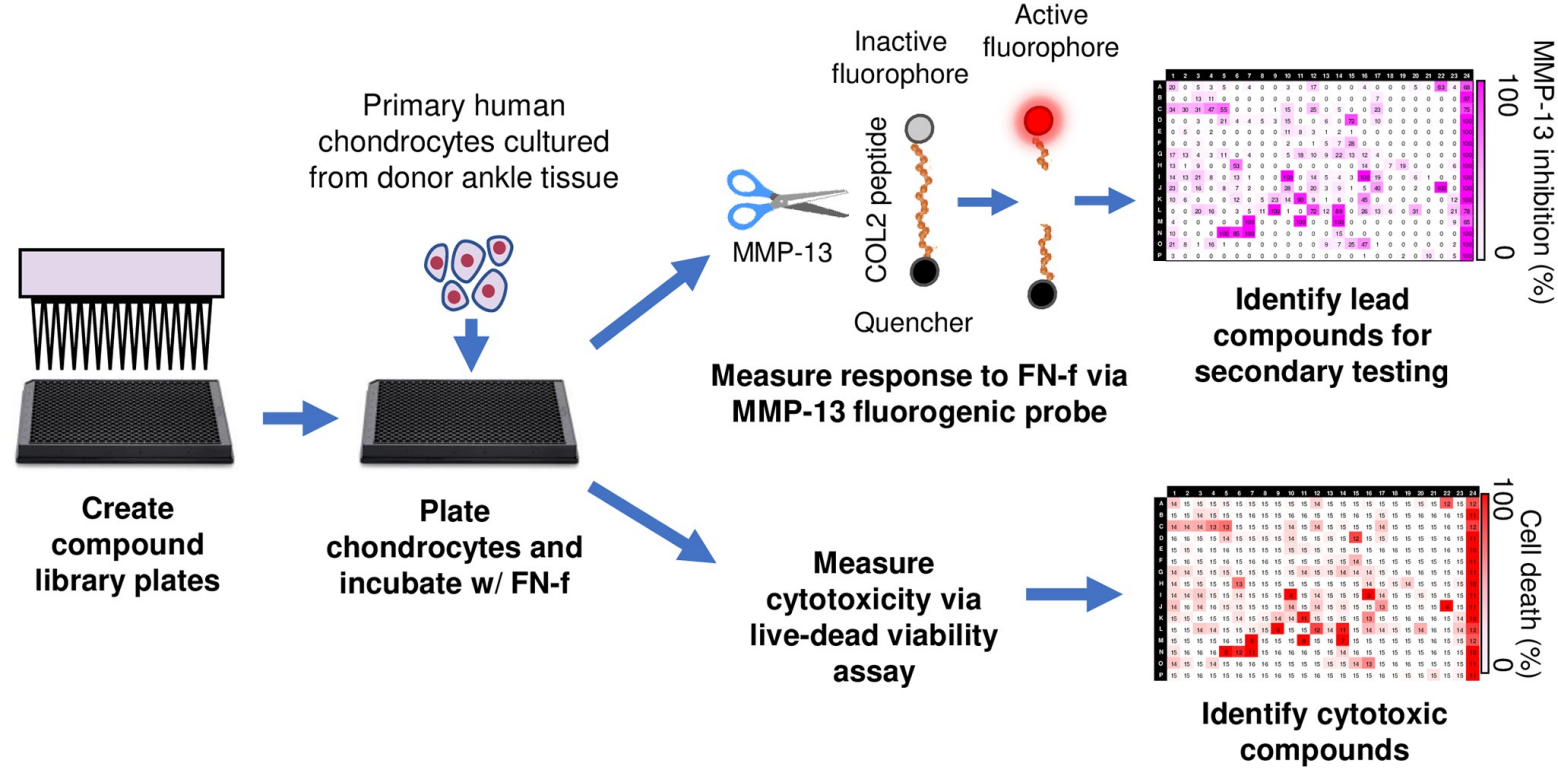
Schematic of high-throughput compound screen protocol for primary human chondrocytes. Compound libraries are “stamped” onto large-scale multiwell plates via an automated liquid handler. Primary human chondrocytes cultured from donor tissue are plated and incubated in compounds for 2hrs. Cells are then treated with a recombinant fibronectin fragment (FN-f) to induce an OA-like phenotype, which includes the expression and secretion of MMP-13. After 24hrs, each compound is tested for cytotoxicity via calcein AM staining. The MMP-13 fluorogenic probe and APMA are then added to the media. APMA converts MMP-13 from its inactive proform to its enzymatically active form, which then cleaves and activates the MMP-13 fluorogenic probe. MMP-13 activity in each well is measured via a fluorescence microplate reader. Each compound is assigned an inhibition score based on the MMP-13 fluorescent readout to identify hits.

**Fig 2:**
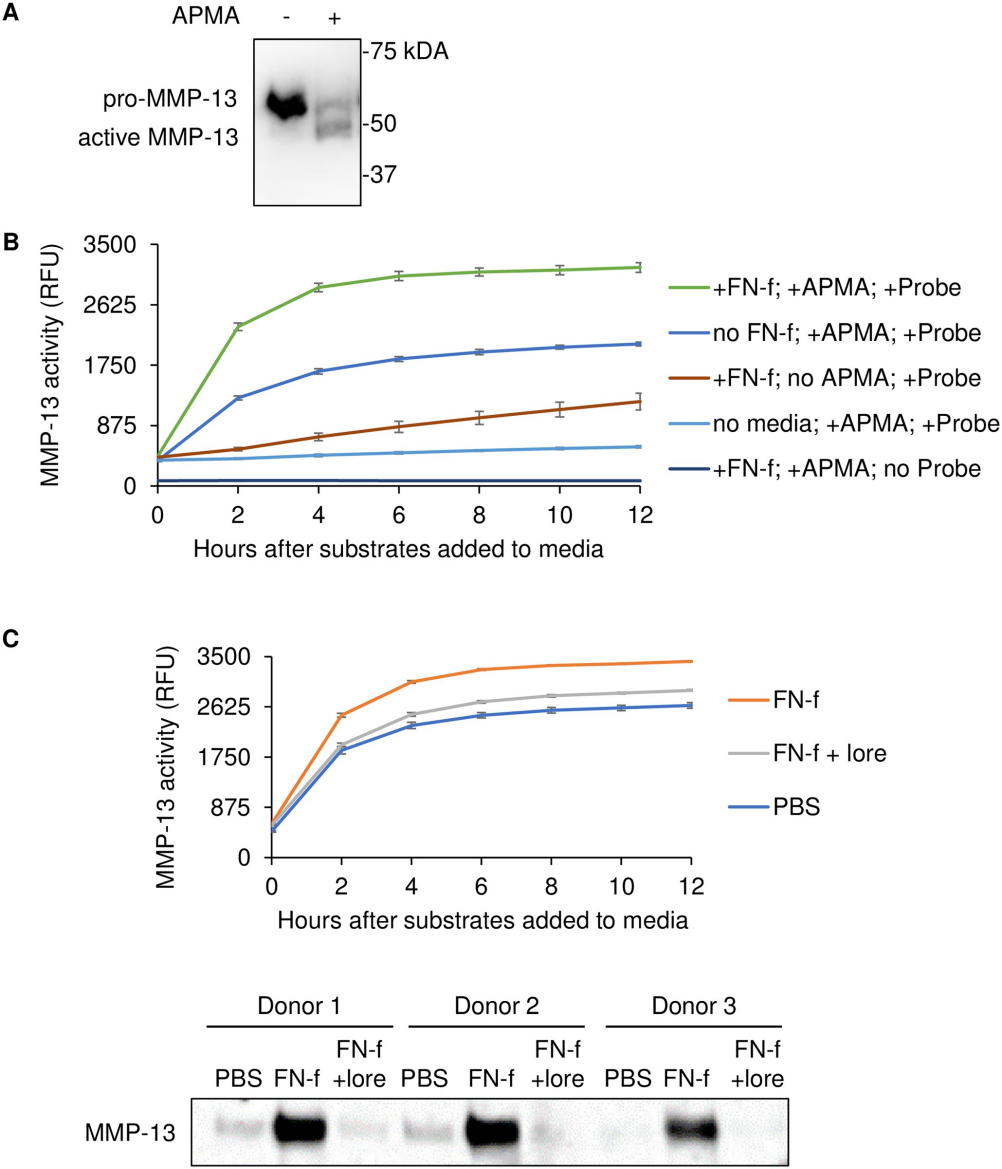
Fluorescent MMP-13 activity probe validation. Primary human chondrocytes from tissue donors (n = 3) were cultured and treated with PBS or FN-f for 24hrs. Media samples were collected and inoculated with or without APMA and/or a fluorescent MMP-13 activity probe substrate. Fluorescence was measured via a microplate spectrophotometer. **A)** Immunoblot staining for MMP-13 from media samples collected from FN-f treated cells followed with or without 1hr APMA treatment. **B)** Quantification of MMP-13 probe fluorescence with indicated treatments. **C)** Primary human chondrocytes from tissue donors (n = 3) were cultured and treated for 2hrs with or without lorecivivint (1 μM). Cells were then treated with PBS or FN-f for 24hrs. Media samples were collected and inoculated with APMA and/or a fluorescent MMP-13 activity probe (upper panel) or immediately used for immunoblotting and MMP-13 staining (lower panel).

To find small molecule inhibitors of OA catabolic signaling, we developed a high-throughput compound screening protocol that used primary human chondrocytes treated with FN-f as a stimulus and MMP-13 as a readout (Fig 1). To measure MMP-13 production using an HTS platform, we developed a fluorogenic probe that measured MMP-13 activity in cell culture media via Förster resonance energy transfer (FRET). This probe consisted of a fluorophore and quencher molecule attached to a small peptide sequence containing the type-II collagen cleavage site that fluoresces in response to targeted enzymatic cleavage by active MMP-13. To test this probe, human articular chondrocytes were first stimulated to secrete MMP-13 via 24hr treatment with FN-f. Because we were interested in compounds that inhibited MMP production and not just MMP activation, conditioned media was incubated with the MMP-13 probe, along with 4-aminophenylmercuric acetate (APMA) that converts all MMP-13 present to its enzymatically active form (Fig 2A). Compared to unstimulated cells, FN-f stimulation resulted in significantly more MMP-13 detection by the probe when APMA was also present (Fig 2B). Next, to test the robustness of the screening assay, human chondrocytes were pre-treated with lorecivivint, a small molecule inhibitor of chondrocyte catabolic signaling that is in phase 3 clinical trials as a disease-modifying drug for knee OA [20]. MMP-13 detection by the probe in FN-f stimulated cells was significantly reduced when chondrocytes were pre-treated with lorecivivint (Fig 2C). Z-prime factor (Z’) was calculated at each timepoint to determine assay robustness, and Z’ > 0.5 was considered robust [27] (S1 Fig). 8hrs after the MMP-13 probe and APMA were added to the media, Z’ was 0.78, indicating strong assay robustness. Accordingly, 8hrs was chosen as the endpoint for future compound screening. Overall, these results demonstrated that the MMP-13 probe can detect MMP-13 secreted by chondrocytes into cell culture media and confirmed that the screening assay was robust.

### Screening of 1280 pharmacologically active compounds

The 1280 LOPAC compound set was screened for inhibition of FN-f induced MMP-13 production in normal human chondrocytes (S1 File). Controls included chondrocytes treated with PBS in place of FN-f to measure the basal production of MMP-13 and chondrocytes treated with FN-f and with DMSO in place of the small molecules to measure maximal MMP-13 production. Compounds were tested at a final concentration of 10 μM. Less than 50% MMP-13 inhibition was noted with 1231 of the LOPAC compounds while 49 achieved over 50% MMP-13 inhibition. Table 1 lists 20 compounds that achieved over 80% MMP-13 inhibition, including seven compounds that completely eliminated FN-f induced MMP-13. The targets of the top 20 “hits” included purogenic (P) 2 receptors P2Y and P2X (suramin sodium salt, NF023, and reactive blue 2), NFκB pathway member IκBα (Bay 11–7085 and Bay 11–7082), STAT3 (stattic), protein phosphatase 1 and protein phosphatase 2A (cantharidic acid), and CDK1 (CGP-74514A hydrochloride).

### Cytotoxicity screening

To exclude the possibility that reductions in MMP-13 production were caused by cell death, LOPAC compounds were also screened for cytotoxicity, in parallel with the MMP-13 inhibitor screen, by staining chondrocytes with calcein AM, 24hrs after compound treatment. As shown in Table 1, this assay revealed that several LOPAC compounds which exhibited high MMP-13 inhibition also resulted in cell death, including the STAT3 inhibitor stattic, and IκBα inhibitors Bay 11–7085 and Bay 11–7082.

### Secondary testing and confirmation

Three LOPAC compounds were selected for secondary testing in dose response experiments to confirm the results of the high throughput screens: stattic, BAY 11–7085, and a CDK-1 inhibitor. Although CGP-74514A used in the LOPAC screen was classified as a CDK1-specific inhibitor, previous literature suggested this compound acted as a pan-CDK inhibitor [28]. Accordingly, we substituted RO-3306 for CGP-74514A in the secondary screen, which has been demonstrated to be highly selective for CDK1 [28].

**Fig 3:**
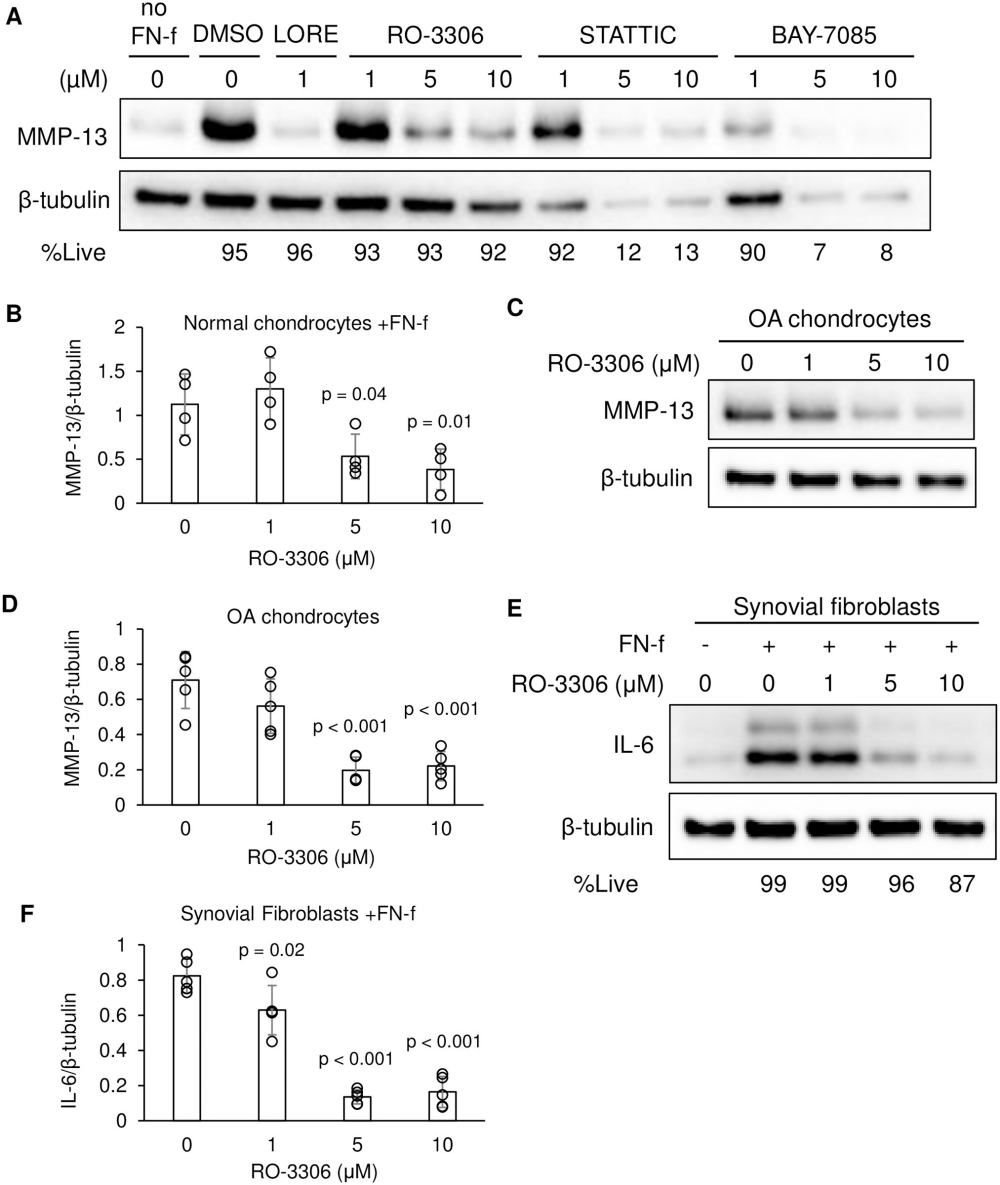
Secondary testing of selected LOPAC compounds. Primary human articular chondrocytes from ankle donors were cultured and treated for 2hrs with selected compounds at the concentrations shown (μM). Cells were then treated with FN-f (1 μM) for 24hrs. “no FN-f” indicates PBS was used instead of FN-f. Media and whole cell lysates were then collected for immunoblot analysis. **A)** Representative immunoblot stained for MMP-13 (from media) and tubulin (from whole cell lysates). LORE = lorecivivint. %Live is the percentage of live cells measured via live-dead assay 24hrs after the indicated treatment. **B)** Immunoblot quantification of RO-3306 effects on MMP-13 from normal chondrocytes treated with FN-f (n = 4, mean±SD). P-values were calculated relative to 0 μM treatment via one-way ANOVA and Dunnett’s test. **C)** Representative immunoblot from media and whole cell lysates of OA knee articular chondrocytes treated with RO-3306 for 24hrs. **D)** Quantification of immunoblot from OA chondrocytes (n = 5, mean±SD). P-values were calculated relative to 0 μM treatment via one-way ANOVA and Dunnett’s test. **E)** Representative immunoblot from media and whole cell lysates of synovial fibroblasts treated with RO-3306 for 24hrs. **F)** Quantification of immunoblot from OA synovial fibroblasts (n = 5, mean±SD). P-values were calculated relative to 0 μM treatment via one-way ANOVA and Dunnett’s test.

Follow-up testing to confirm MMP-13 inhibition was performed on human ankle articular chondrocytes treated with FN-f as in the primary screen except for the use of standard sized cell culture wells and analysis of MMP-13 by immunoblot. Stattic, BAY 11–7085, RO-3306, and lorecivivint inhibited FN-f induced MMP-13 production (Fig 3A). However, significant loss of tubulin, used as a protein loading control on the immunoblots, was observed with stattic and BAY 11–7085 treatments at doses greater than 1 μM, indicating a potential loss of cellular protein resulting from cell death. Live-dead cell analysis using calcein AM and ethidium homodimer-1 staining confirmed that stattic and BAY 11–7085 were cytotoxic at 5 and 10 μM, but RO-3306 and lorecivivint were not (Fig 3A and S2 Fig). Quantification of immunoblots performed on FN-f treated chondrocytes obtained from four independent donors confirmed that RO-3306 inhibited FN-f induced MMP-13 in a dose-dependent manner (Fig 3B).

Since RO-3306 demonstrated significant inhibition of FN-f induced MMP-13 with low cytotoxicity, it was chosen for additional follow-up testing using human knee articular chondrocytes obtained from OA donors to examine if targeting CDK1 inhibited MMP-13 production. Our lab has previously shown that the response to FN-f is not joint-specific with activation of similar signaling pathways in knee and ankle chondrocytes [29]. Importantly, since these chondrocytes were from an OA joint, they were not treated with FN-f and so only basal (unstimulated) MMP-13 production was measured. Treatment with RO-3306 resulted in significantly less MMP-13 production by OA knee chondrocytes in a dose dependent manner (Fig 3C and 3D).

Next, we tested RO-3306 on synovial fibroblasts isolated from the synovial tissue obtained from OA donors and stimulated with FN-f to determine if inhibiting CDK1 could inhibit production of the proinflammatory cytokine IL-6. Although the synovial fibroblasts were from OA tissue, the basal levels of IL-6 produced were quite variable but were consistently increased by FN-f (unpublished data). IL-6 is involved in signaling pathways that can induce the expression of matrix metalloproteinases (MMPs) and has been found in the synovial fluid of OA patients [30]. Similar to MMP-13 in chondrocytes, RO-3306 reduced FN-f-stimulated IL-6 production by synovial fibroblasts in a dose dependent manner (Fig 3E and 3F). Additionally, tubulin levels remained equal, and live-dead assay confirmed that RO-3306 was not cytotoxic in synovial fibroblasts at the concentrations tested.

### Dose range screening of kinase inhibitors

**Fig 4:**
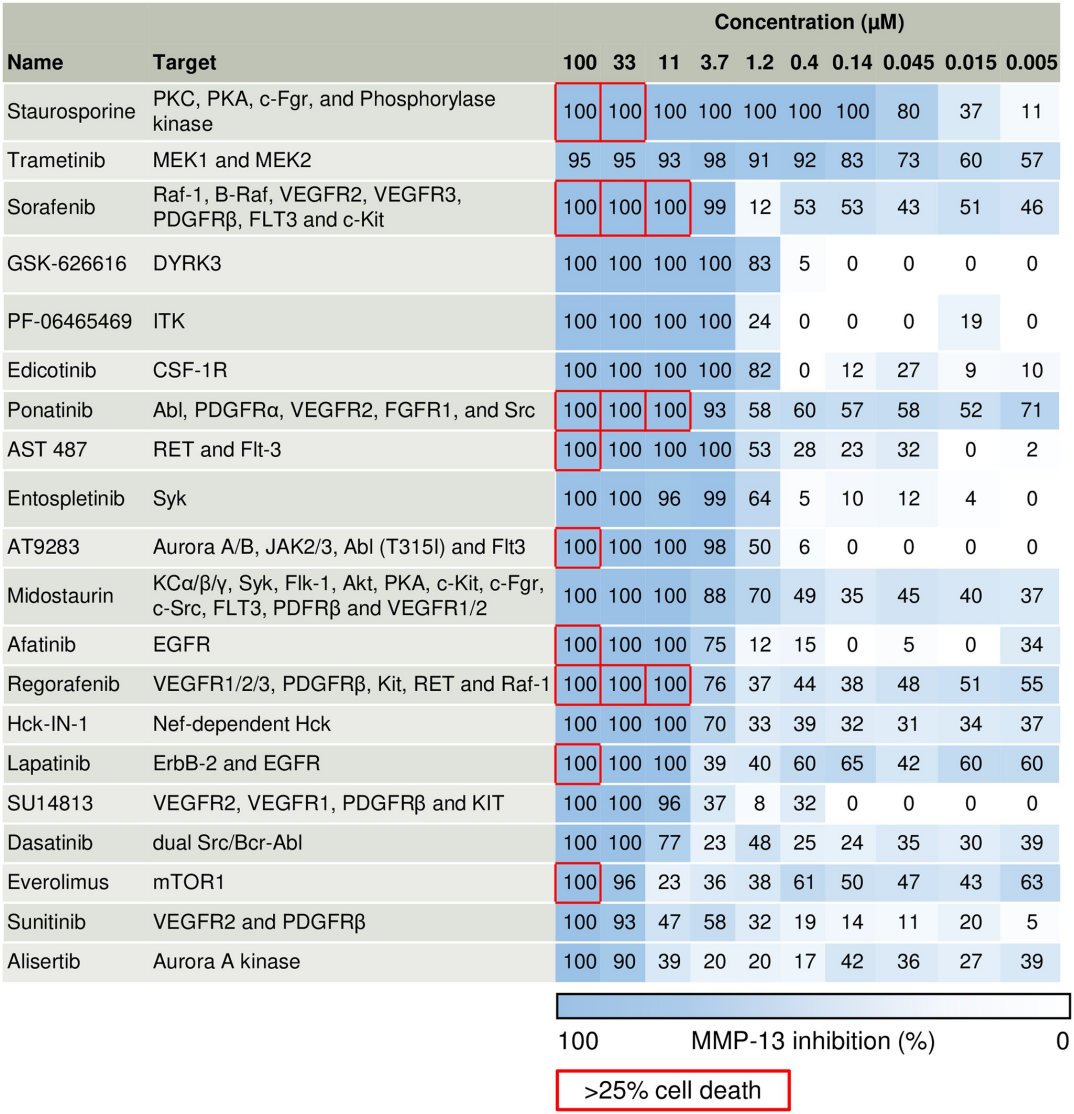
Top 20 kinase inhibitors with the strongest inhibition of MMP-13. Names of kinase inhibitors and the target kinase or pathways are shown. Numbers shaded blue represent % inhibition of FN-f induced section of MMP-13. Red squares represent compound concentrations that resulted in more than 25% cell death after 24hrs.

Next, we screened a custom library of 64 kinase inhibitors for inhibition of FN-f induced MMP-13 secretion and cytotoxicity (Fig 4 and S2 File). To avoid the confounding effects of cytotoxicity caused by using only a single concentration, we tested ten concentrations ranging from 5 nM to 100 μM. Of the 64 compounds screened, staurosporine (inhibits PKC, PKA), trametinib (inhibits MEK1/2), edicotinib (inhibits CSF-1R), and GSK-626616 (inhibits DYRK3) exhibited the strongest inhibition of MMP-13 production at relatively low concentrations (≤1.2 μM) and with low cytotoxicity.

### Secondary testing and confirmation of kinase inhibitor screen

**Fig 5:**
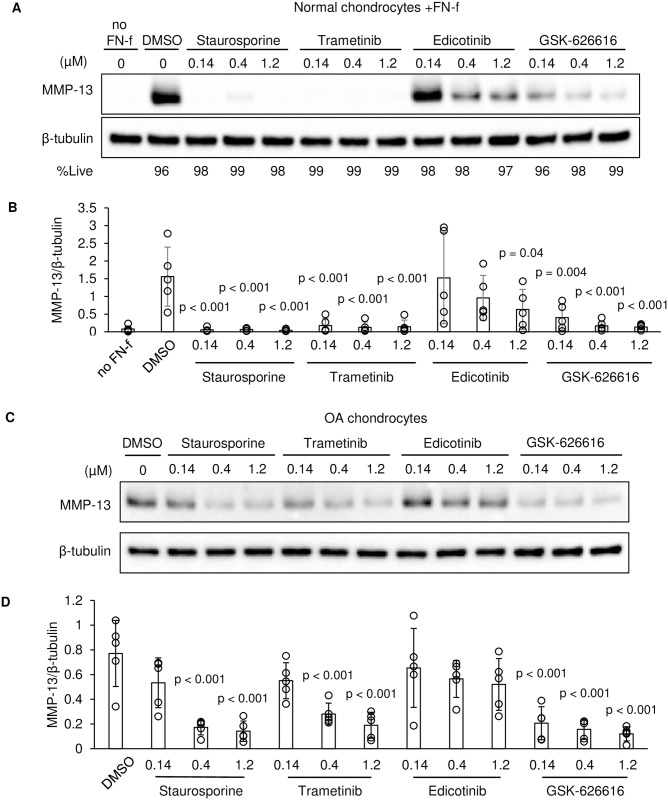
Secondary testing of selected kinase inhibitors in chondrocytes. **A)** Representative immunoblots from media and whole cell lysates of primary articular chondrocytes treated with FN-f and indicated compounds for 24hrs. %Live is the percentage of live cells measured via live-dead assay 24hrs after the indicated treatment. **B)** Quantification of immunoblots from normal human chondrocytes (n = 5, mean±SD). P-values were calculated relative to DMSO via one-way ANOVA and Dunnett’s test. **C)** Representative immunoblot from media and whole cell lysates of OA knee articular chondrocytes treated with indicated compounds for 24hrs. **D)** Quantification of immunoblots from OA chondrocytes (n = 5, mean±SD). P-values were calculated relative to DMSO via one-way ANOVA and Dunnett’s test.

Secondary testing of the kinase set was performed via immunoblot using FN-f treated normal human chondrocytes obtained from 3 independent donor ankle tissues. Immunoblots confirmed staurosporine, trametinib, and GSK-626616 significantly inhibited FN-f induced MMP-13 in chondrocytes (Fig 5A and 5B). Edicotinib inhibited MMP-13 with varying efficacies in 5 different tissue donors but was not statistically significant compared to a DMSO control group. Live-dead analysis confirmed that these compounds were not cytotoxic in chondrocytes at the concentrations tested (Fig 5A and S3 Fig).

Follow-up testing of these compounds in human knee articular chondrocytes obtained from OA donors confirmed the ability of staurosporine, trametinib, and GSK-626616, but not edicotinib, to inhibit basal MMP-13 production in the diseased cells (Fig 5C and 5D).

**Fig 6:**
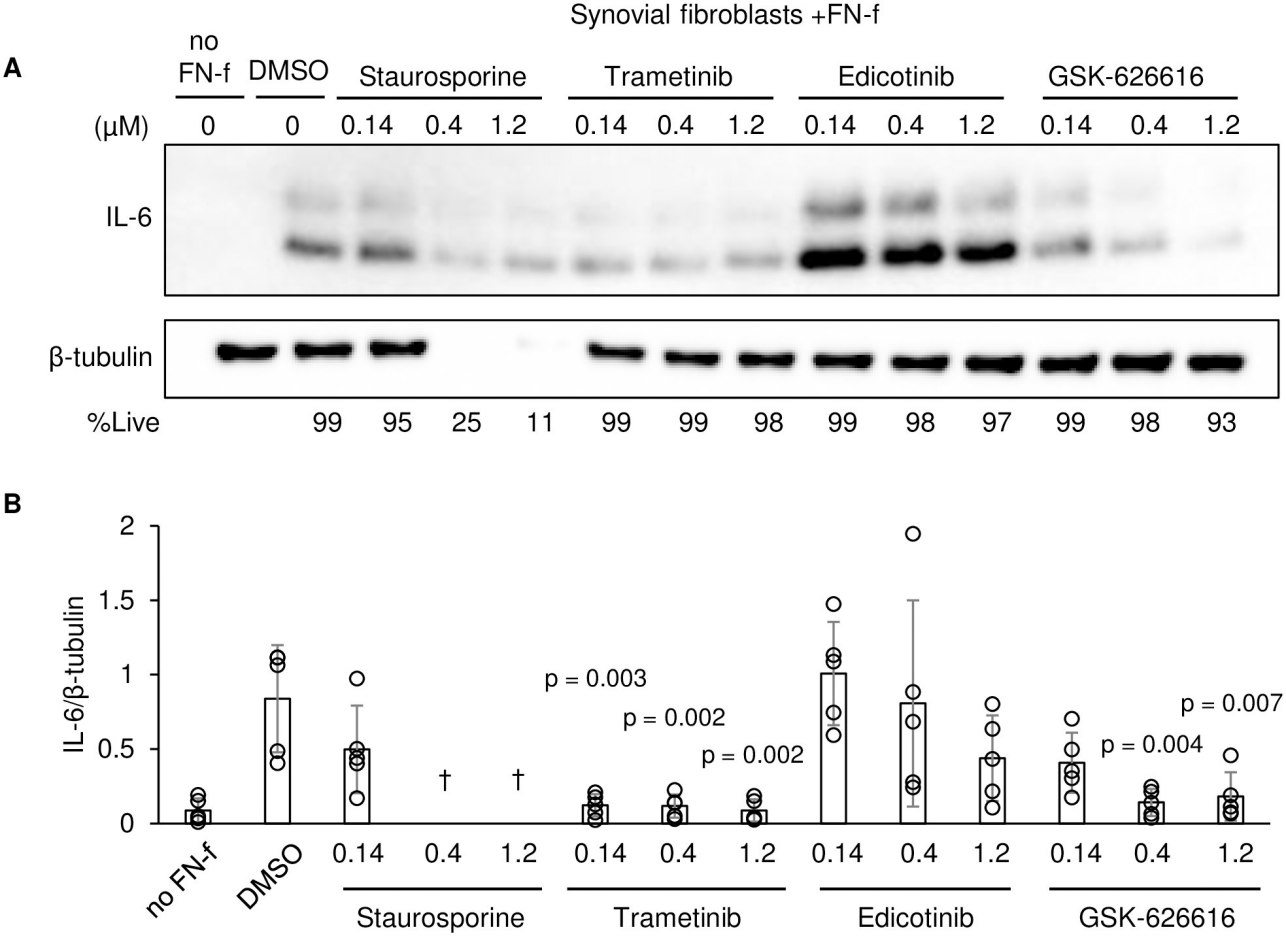
Secondary testing of selected kinase inhibitors in synovial fibroblasts. **A)** Representative immunoblots from media and whole cell lysates of OA synovial fibroblasts treated with indicated compounds for 24hrs. **B)** Quantification of immunoblots from OA synovial fibroblasts (n = 5, mean±SD). † = not quantified due to cytotoxicity. P-values were calculated relative to DMSO via one-way ANOVA and Dunnett’s test.

Next, we tested these kinase inhibitors on synovial fibroblasts treated with FN-f to determine if they could also inhibit IL-6 production (Fig 6A and 6B). Notably, the PKC inhibitor staurosporine was cytotoxic in synovial fibroblasts, demonstrating cell-specific cytotoxicity that did not occur in chondrocytes. Trametinib, which is a MEK1/2 inhibitor, significantly reduced IL-6 production without cytotoxicity at all concentrations tested. The CSF-1R inhibitor edicotinib demonstrated considerable donor-specific variability on OA synovial fibroblast IL-6 secretion, including increased IL-6 secretion in some treated donors. The DYRK inhibitor GSK-626616 demonstrated strong IL-6 inhibition but with mild cytotoxicity at 1.2 μM.

## Discussion

We developed a unique cell-based high-throughput screening platform that utilizes human chondrocytes to discover potential OA disease modifying small molecules. Of 1344 compounds tested in two screens, 20 demonstrated >80% inhibition of MMP-13 production by chondrocytes using FN-f stimulation as an *in vitro* model of OA. Of these, 5 were determined to also have low cytotoxicity and high efficacy in both FN-f treated normal human chondrocytes and unstimulated chondrocytes derived from OA donors. These included RO-3306 (CDK1i), staurosporine (PKCi), trametinib (MEK1 and MEK2i), GSK-626616 (DYRK3i), and edicotinib (CSF-1Ri). Follow-up testing confirmed all but one of these compounds (edicotinib) inhibited basal MMP-13 production by OA chondrocytes. Compounds that can also inhibit the proinflammatory activity of synovial fibroblasts would have an advantage over those that only affect chondrocytes. RO-3306, trametinib, and GSK-626616 were found to reduce IL-6 production by OA synovial fibroblasts stimulated with FN-f without significant cytotoxicity supporting the robustness of our HTS platform in detecting potential disease-modifying compounds for OA treatment.

Other high-throughput screening platforms for OA have been developed. This includes the screening platform used to discover lorecivivint that utilized a colon cancer cell line with high Wnt activity and a TCF/LEF-reporter based assay to discover compounds targeting the Wnt signaling pathway [31]. Secondary screens were done in chondrogenesis assays and assays of catabolic activity using human bone marrow derived mesenchymal stem cells. A limitation to this HTS approach is the focus on the discovery of compounds targeting the Wnt pathway, which may or may not be a critical pathway in OA or perhaps in a subset of individuals with OA. Another HTS approach used a chondrosarcoma FoxO1-luciferase reporter cell line to discover compounds that induce FoxO1 with follow-up testing in human OA chondrocytes, meniscus cells and synovial fibroblasts [32]. The top hits discovered with this assay were histone deacetylase (HDAC) inhibitors. Jundi et al [33] reported using engineered cartilage constructs in a single-impact compression model with readouts of matrix degradation and cell stress using glycosaminoglycan and lactate dehydrogenase assays respectively. Limitations included the development of the assay using bovine tissue rather than human and use of a 48 well format which reduces the number of compounds that can be screened in each assay. It is also not clear how well a single impact given to cartilage constructs models the complex signaling that occurs in OA.

The goal of our HTS was to take a different approach from other OA small molecule screening platforms by using primary human chondrocytes and a stimulus, FN-f, that activates cell signaling pathways that regulate expression of a host of OA mediators including cytokines, chemokines, and matrix degrading enzymes found in OA [11, 13, 34]. As a readout, we used a probe to measure production of MMP-13, a key matrix degrading enzyme in OA cartilage, with the knowledge that many of the signaling pathways regulating MMP-13 are in common with those regulating other OA catabolic mediators.

Our assay results were consistent with prior work that tested the effects on chondrocyte MMP production using some of the same inhibitors found in the HTS. Inhibitors to MEK1/2 and PKC have been previously shown to block FN-f-induced MMP production using a 100 kDa FN-f [35, 36], and lorecivivint was shown to block IL-1β-stimulated MMP production [20]. CSF-1R is overexpressed in the synovium of patients with severe synovitis and a CSF-1R antibody was found to protect bone and cartilage in the collagen-induced mouse model of rheumatoid arthritis [37]. However, our results using the CSF-1R inhibitor edicotinib suggests it may not be an optimal treatment for OA since it did not reduce basal production of MMP-13 by OA chondrocytes and was much less effective than other compounds in reducing IL-6 production by synovial fibroblasts stimulated with FN-f.

To our knowledge, CDK1 has not been previously examined in chondrocytes or in the context of OA. However, bioinformatics analysis of genes dysregulated in human OA cartilage identified the CDK1 inhibitor 1A (p21) as a key downregulated OA gene [38] suggesting loss of CDK1 inhibition from downregulation of p21 could contribute to OA by allowing increased CDK1 activity. According to the OpenTargets database for drug target identification, CDK1 has a druggable score of >0.7 (0–1 scale) [39], and CDK1 inhibitors are in early phase trials for certain malignancies. We searched CDK1 in OATargets [40], a database of genes associated with OA joint damage in animal models, and in the Musculoskeletal Knowledge Portal [41] and found that a *Cdk1* gene variant has been associated with bone density but not OA [42]. Additionally, CDK1 promotes β-catenin activity through PDK1 [43] and CDK1/2 activity has been shown to promote cell senescence in response to DNA damage [44]. Both cellular senescence and activation of Wnt signaling, including increased β-catenin activity, have been shown to contribute to OA [45, 46]. Given that the CDK1-specific inhibitor RO-3306 demonstrated potent inhibition of OA signaling that mediates chondrocyte MMP-13 production and synovial fibroblasts IL-6 production *in vitro*, we believe CDK1 should be further investigated as a potential target for OA therapeutics.

DYRK3 has also not been characterized as a gene relevant to OA pathology. However, drugs that target other DYRK family members, such as lorecivivint, have been shown to reduce OA catabolic signaling [20]. Active DYRK3 has been demonstrated to increase mTORC1 activation [47]. mTOR regulates the senescence-associated secretory phenotype (SASP) which has been implicated in OA pathogenesis [46, 48]. Importantly, the compound used for this screen (GSK-626616) that was classified as a DYRK3 inhibitor also has affinity for other DYRK-family members [47]. Therefore, the inhibition of MMP-13 and IL-6 production demonstrated in the screen and follow-up experiments may have been caused by inhibition of one or more DYRK family members. Given that GSK-626616, a pan-DYRK inhibitor, and lorecivivint, a DYRK1A inhibitor, have both demonstrated potent inhibition of OA catabolic signaling, the DYRK family of kinases should continue to be investigated as potential targets for OA treatment.

Overall, the results of the screen and follow-up experiments demonstrate the robustness of our HTS platform in identifying compounds that inhibit human chondrocyte production of the collagenase MMP-13 in both a FN-f induced OA disease model and in secondary screens using OA chondrocytes. Several of the same compounds also inhibit synovial fibroblast IL-6 production. Further studies are needed to test selected inhibitors *in vivo* using preclinical models of OA. Our efforts reported here also lay the foundations for additional compound set screens for hit and novel target discovery campaigns for OA.

## Supporting information

S1 Fig Z-prime factor graph. Average Z’ calculated every hour for 12hrs after the MMP-13 probe and APMA were added to each compound plate. Normal distribution was determined via Shapiro-Wilk test.
(TIF)

S2 Fig Live-cell cytotoxicity assay using selected LOPAC compounds. Green indicates live cells stained with calcein AM. Red indicates dead cells stained with ethidium homodimer-I. Cells were stained 24hrs. after compounds were added to the cell culture media.
(TIF)

S3 Fig Live-cell cytotoxicity assay using selected kinase inhibitors. Green indicates live cells stained with calcein AM. Red indicates dead cells stained with ethidium homodimer-I. Cells were stained 24hrs. after compounds were added to the cell culture media.
(TIF)

S1 Raw images (PDF)

S1 File (XLSX)

S2 File (XLSX)

S3 File (XLSX)
