# Supplementary figures and images for: A novel small molecule screening assay using normal human chondrocytes toward osteoarthritis drug discovery
Source: PLoS One. 2024 Nov 1;19(11):e0308647. doi: 10.1371/journal.pone.0308647 (PMC11530018; doi:10.1371/journal.pone.0308647)

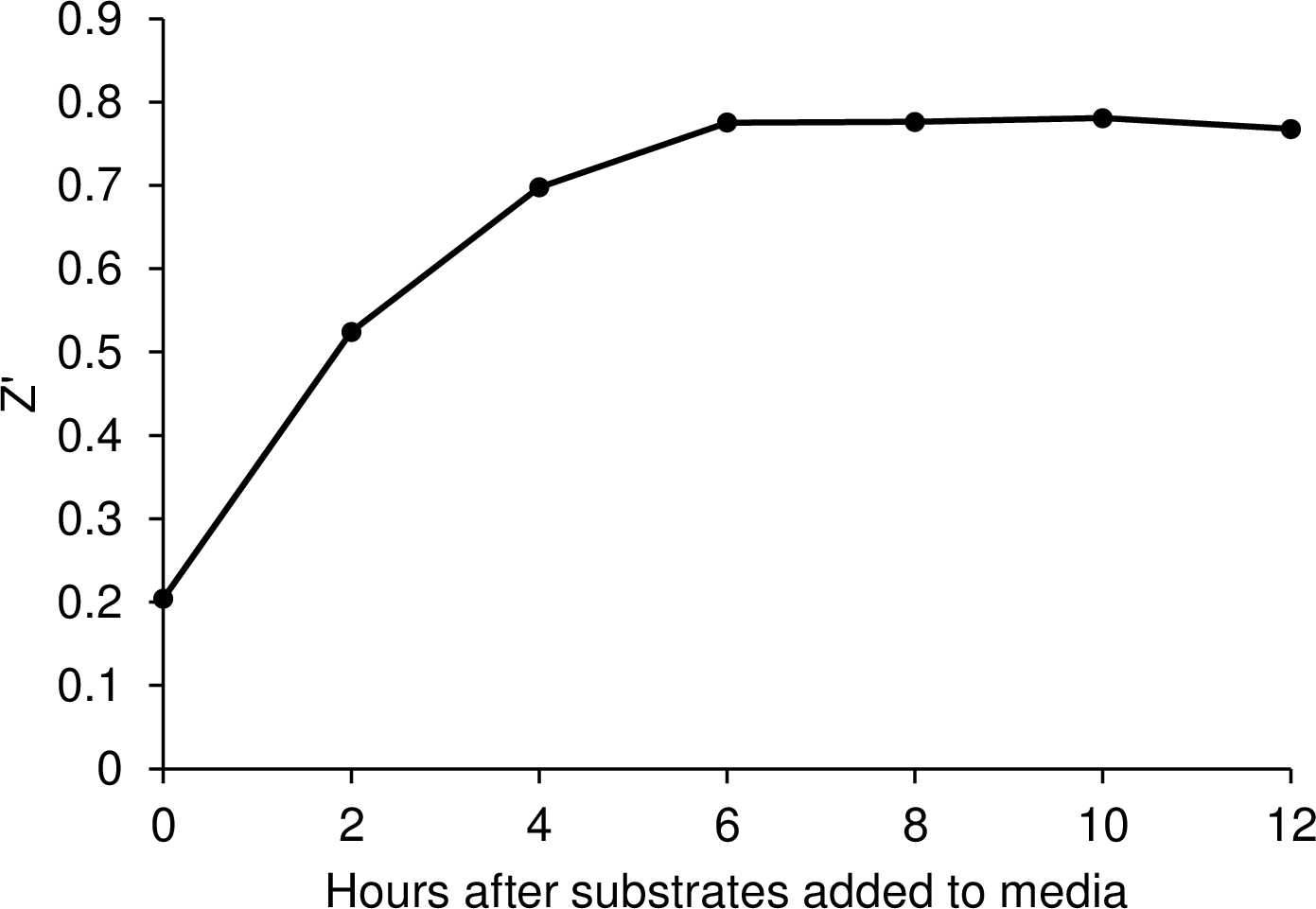

Supplement: S1 Fig — Average Z’ calculated every hour for 12hrs after the MMP-13 probe and APMA were added to each compound plate. Normal distribution was determined via Shapiro-Wilk test. (TIF) [file pone.0308647.s001.tif]

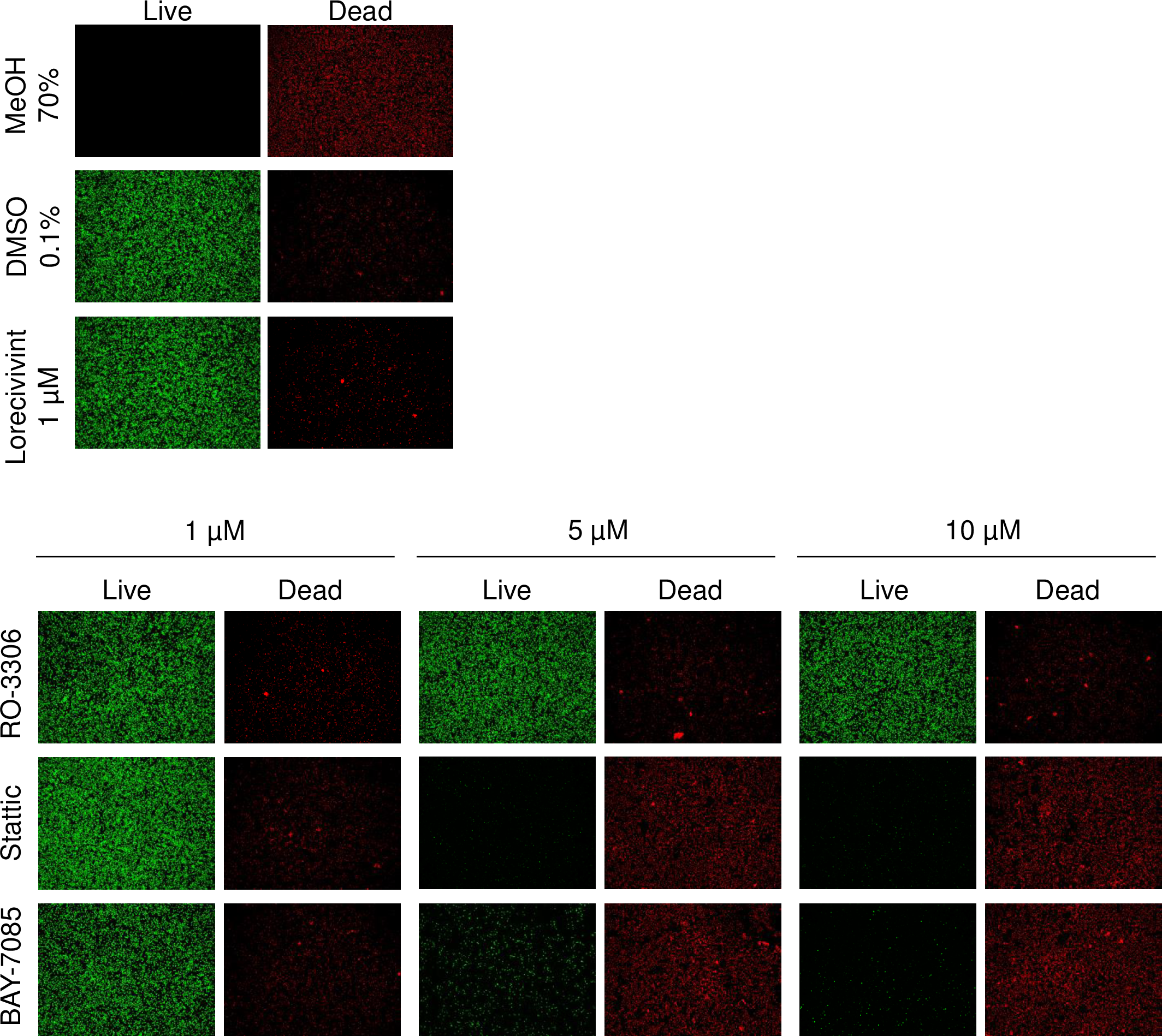

Supplement: S2 Fig — Green indicates live cells stained with calcein AM. Red indicates dead cells stained with ethidium homodimer-I. Cells were stained 24hrs. after compounds were added to the cell culture media. (TIF) [file pone.0308647.s002.tif]

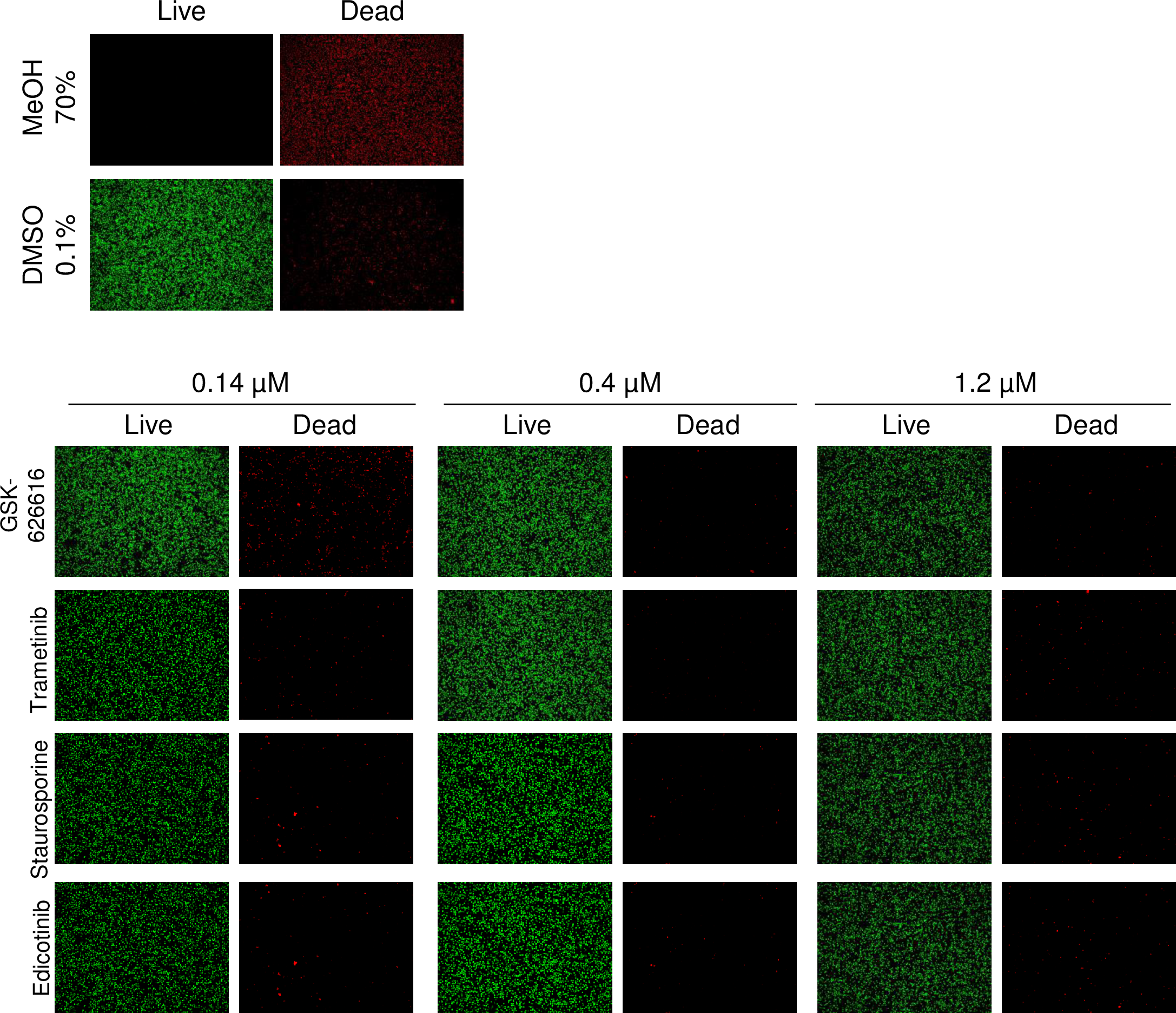

Supplement: S3 Fig — Green indicates live cells stained with calcein AM. Red indicates dead cells stained with ethidium homodimer-I. Cells were stained 24hrs. after compounds were added to the cell culture media. (TIF) [file pone.0308647.s003.tif]
