# Supplementary material for: A novel small molecule screening assay using normal human chondrocytes toward osteoarthritis drug discovery
Source: PLoS One. 2024 Nov 1;19(11):e0308647. doi: 10.1371/journal.pone.0308647 (PMC11530018; doi:10.1371/journal.pone.0308647)
Supplement: S1 Raw images — (PDF) [file pone.0308647.s004.pdf]

Figure 3A – mmp13

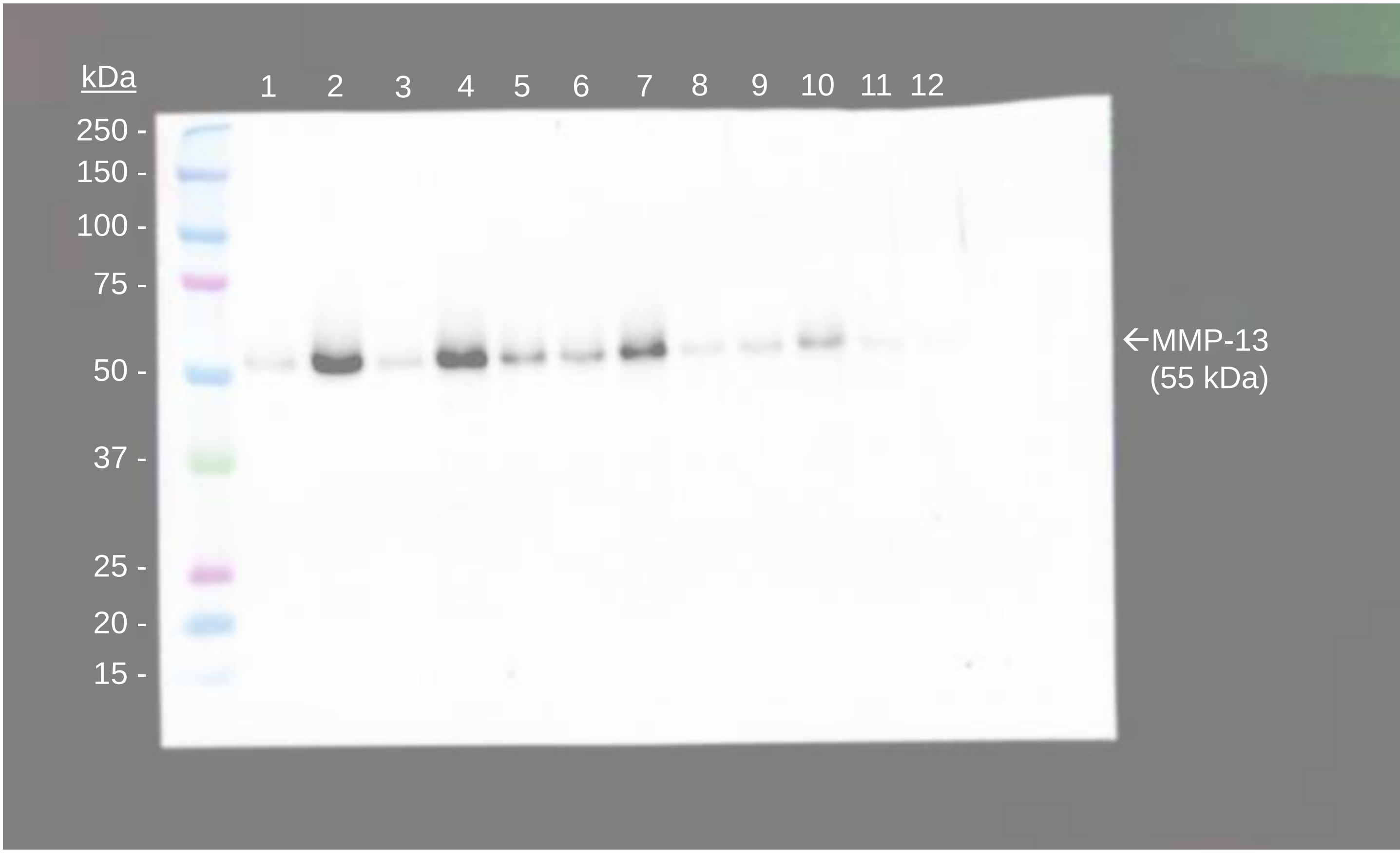

Lanes:

1. No FN-f
2. DMSO
3. Lorecivivint 1 uM
4. RO-3306 1 uM
5. RO-3306 5 uM
6. RO-3306 10 uM
7. STATTIC 1 uM
8. STATTIC 5 uM
9. STATTIC 10 uM
10. BAY-7085 1 uM
11. BAY-7085 5 uM
12. BAY-7085 10 uM

Figure 3A – β-tubulin

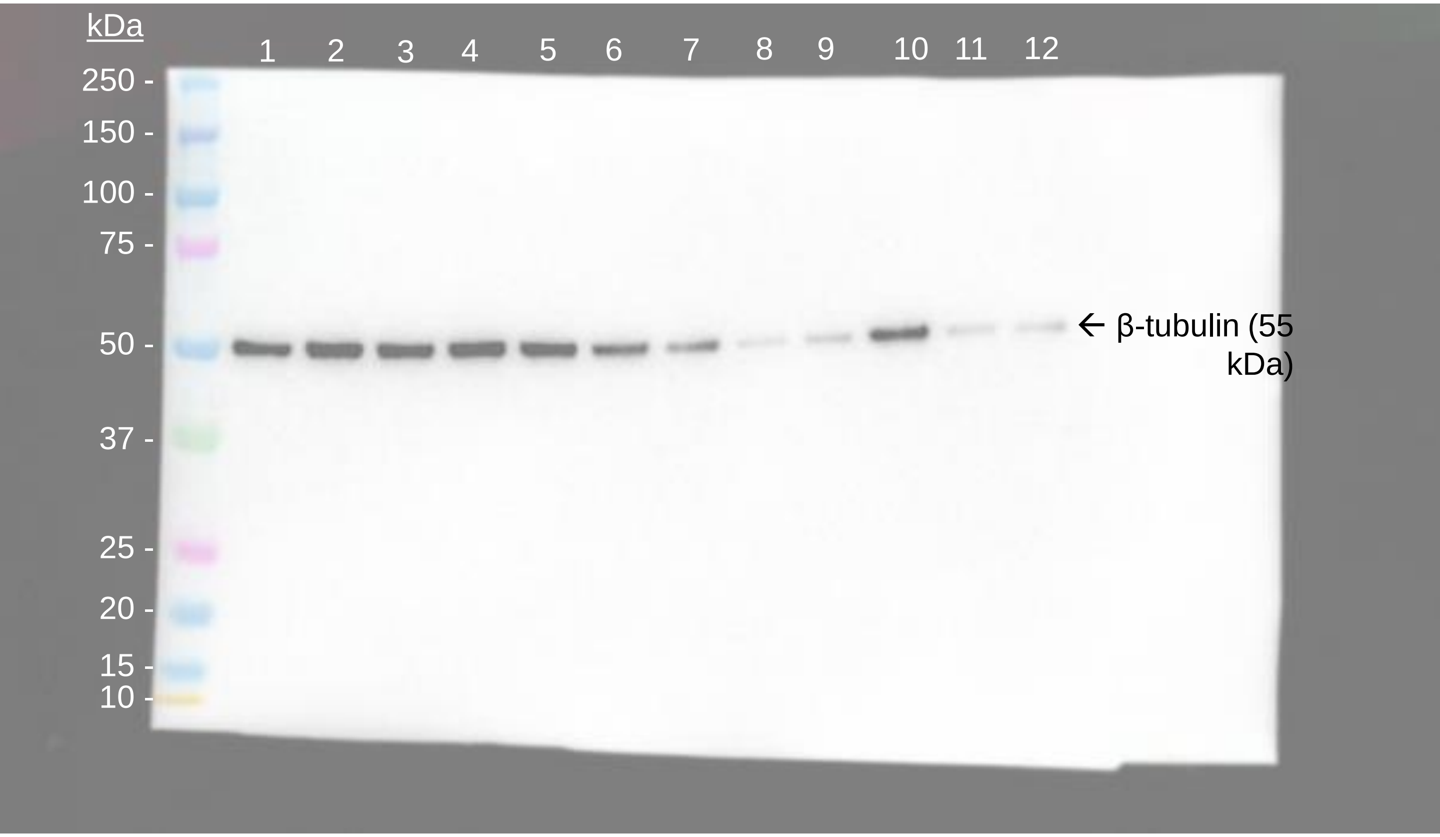

Lanes:

1. No FN-f
2. DMSO
3. Lorecivivint 1 uM
4. RO-3306 1 uM
5. RO-3306 5 uM
6. RO-3306 10 uM
7. STATTIC 1 uM
8. STATTIC 5 uM
9. STATTIC 10 uM
10. BAY-7085 1 uM
11. BAY-7085 5 uM
12. BAY-7085 10 uM

Figure 3C – MMP-13

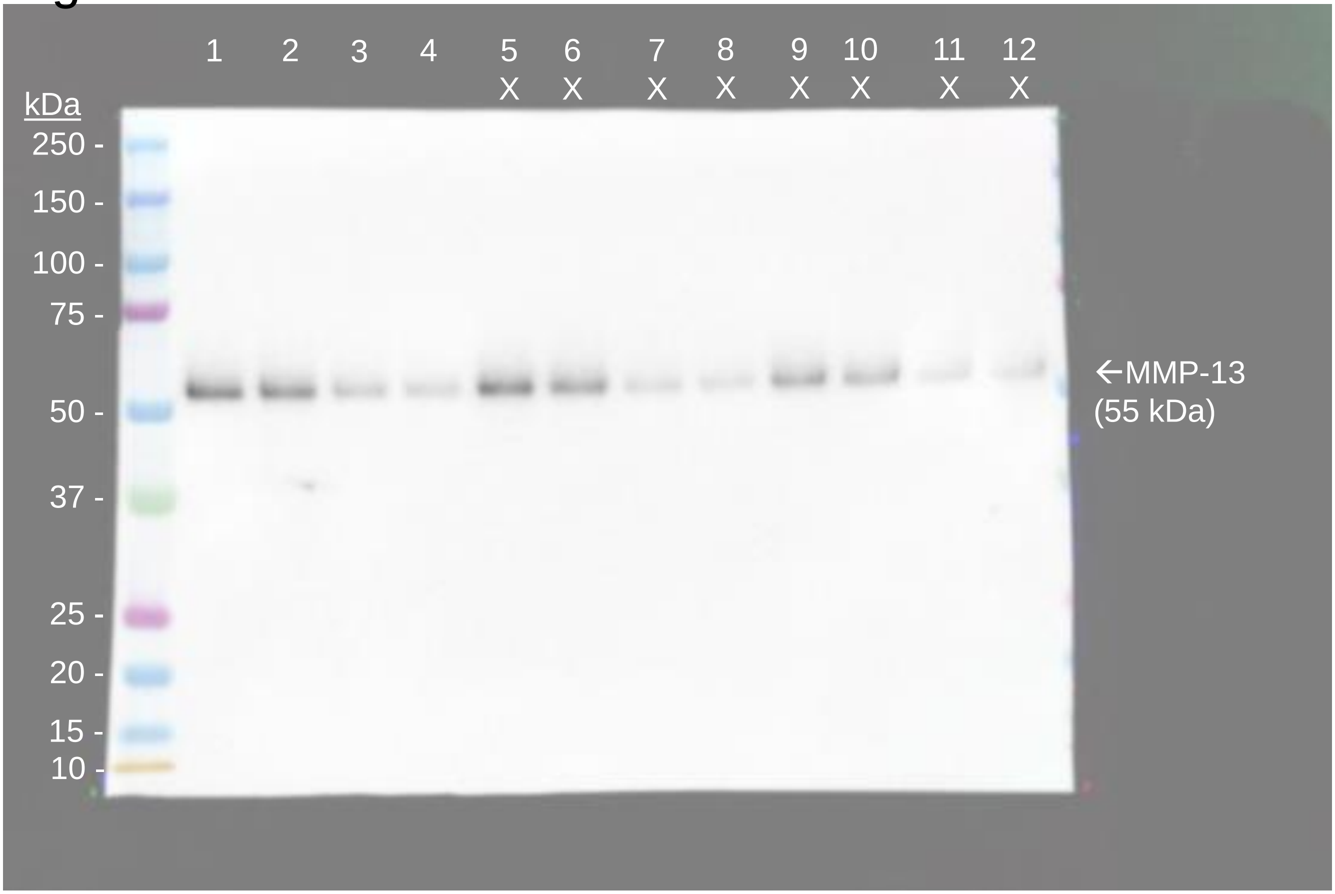

Lanes:

1. OA donor 1 – RO-3306 0 uM
2. OA donor 1 – RO-3306 1 uM
3. OA donor 1 – RO-3306 5 uM
4. OA donor 1 – RO-3306 10 uM
5. OA donor 2 – RO-3306 0 uM
6. OA donor 2 – RO-3306 1 uM
7. OA donor 2 – RO-3306 5 uM
8. OA donor 2 – RO-3306 10 uM
9. OA donor 3 – RO-3306 0 uM
10. OA donor 3 – RO-3306 1 uM
11. OA donor 3 – RO-3306 5 uM
12. OA donor 3 – RO-3306 10 uM

Figure 3C - β-tubulin

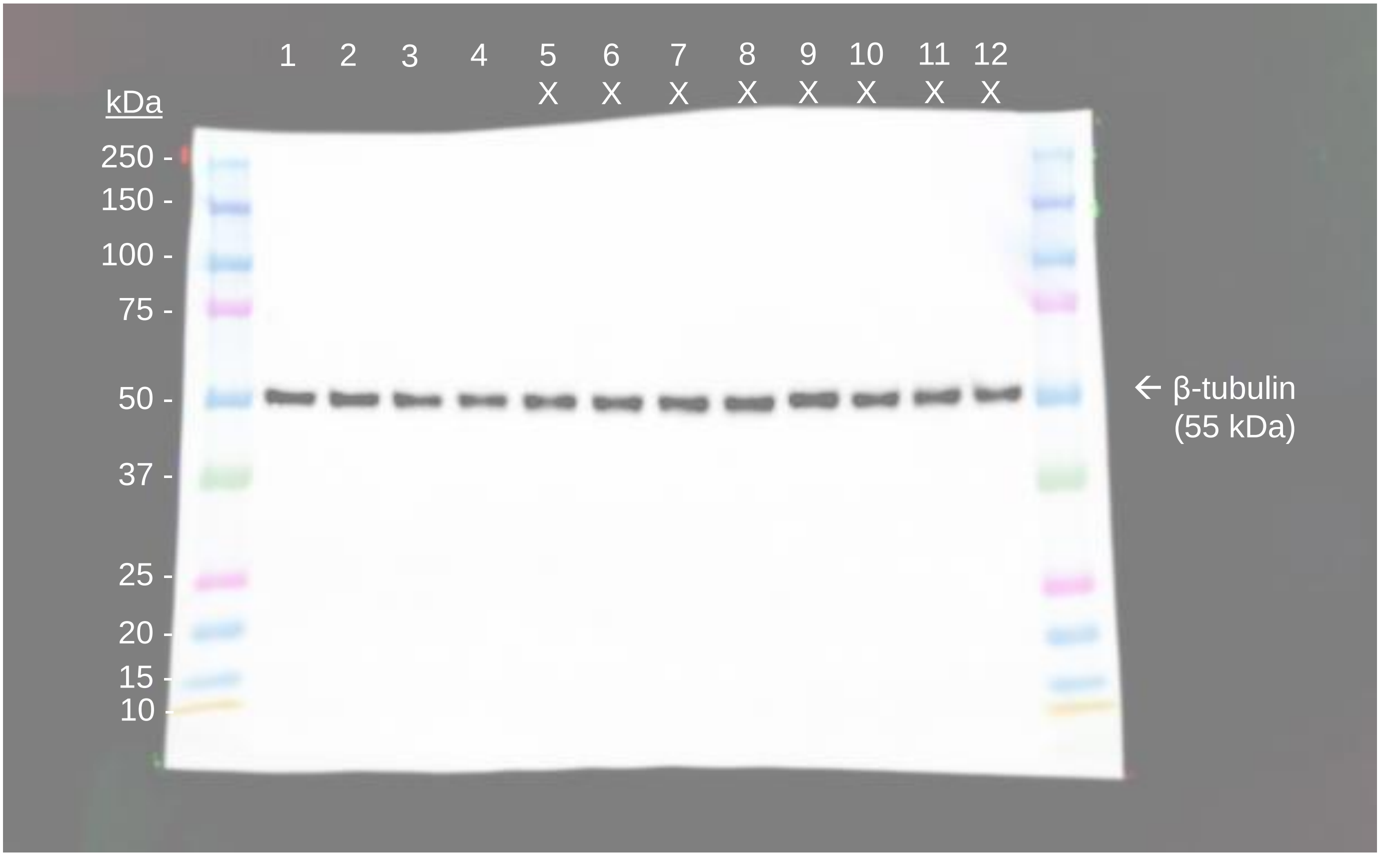

Lanes:

1. OA donor 1 – RO-3306 0 uM
2. OA donor 1 – RO-3306 1 uM
3. OA donor 1 – RO-3306 5 uM
4. OA donor 1 – RO-3306 10 uM
5. OA donor 2 – RO-3306 0 uM
6. OA donor 2 – RO-3306 1 uM
7. OA donor 2 – RO-3306 5 uM
8. OA donor 2 – RO-3306 10 uM
9. OA donor 3 – RO-3306 0 uM
10. OA donor 3 – RO-3306 1 uM
11. OA donor 3 – RO-3306 5 uM
12. OA donor 3 – RO-3306 10 uM

Figure 3E – IL-6

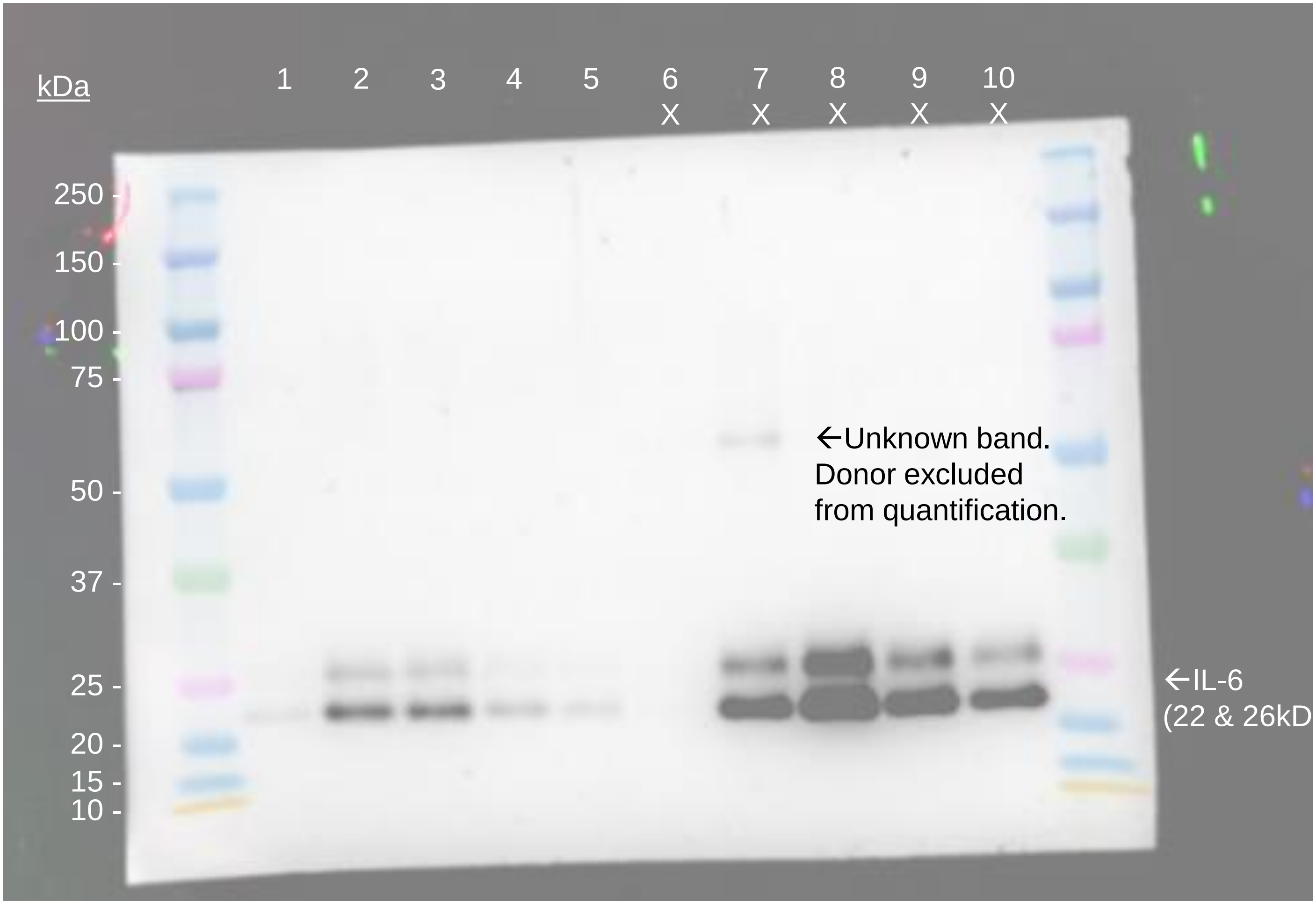

Lanes:

1. Donor 1 – RO-3306 0 uM, no FN-f
2. Donor 1 – RO-3306 0 uM
3. Donor 1 – RO-3306 1 uM
4. Donor 1 – RO-3306 5 uM
5. Donor 1 – RO-3306 10 uM
6. Donor 2 – RO-3306 0 uM, no FN-f
7. Donor 2 – RO-3306 0 uM
8. Donor 2 – RO-3306 1 uM
9. Donor 2 – RO-3306 5 uM
10. Donor 2 – RO-3306 10 uM

Figure 3E – β-tubulin

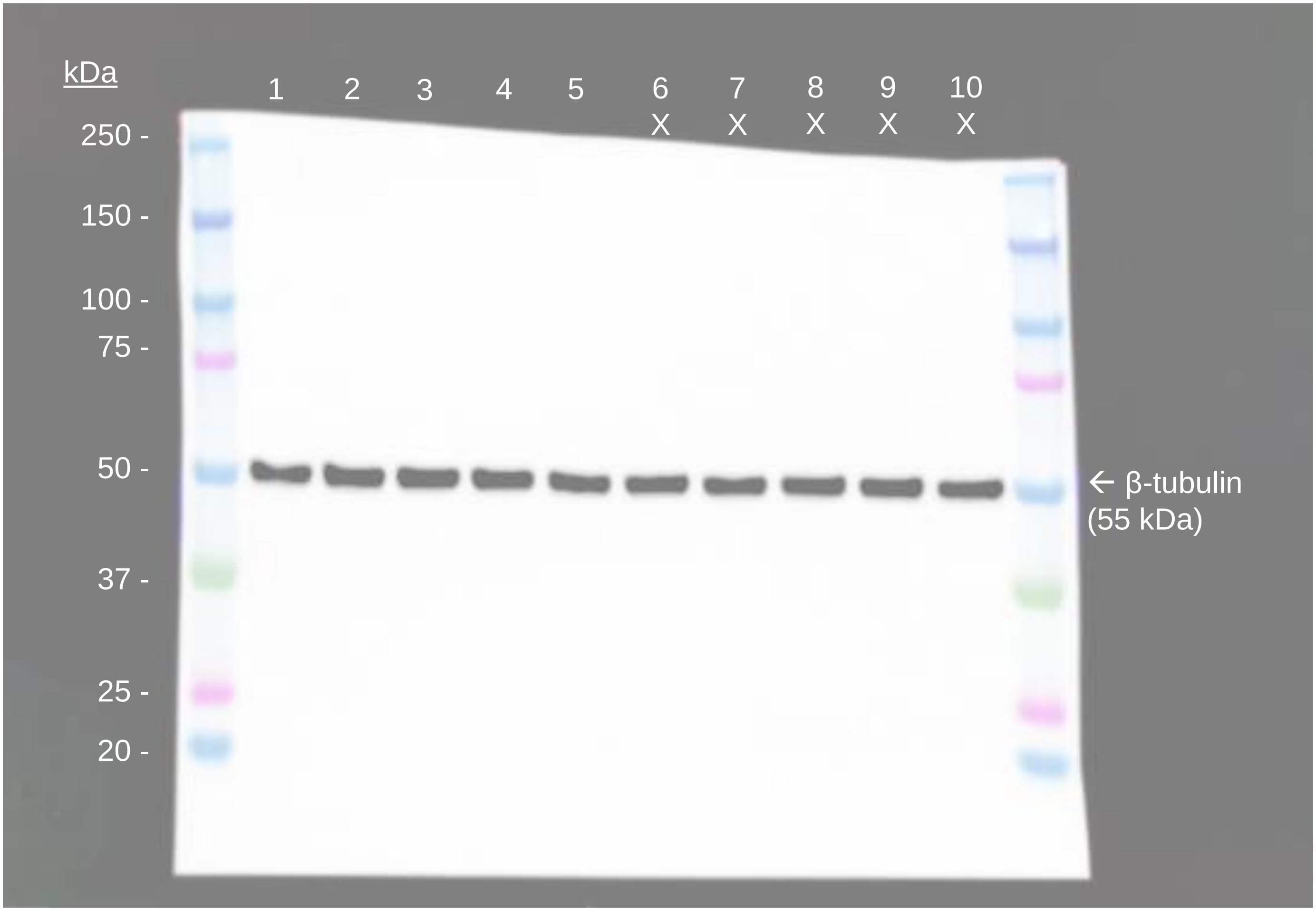

Lanes:

1. Donor 1 – RO-3306 0 uM, no FN-f
2. Donor 1 – RO-3306 0 uM
3. Donor 1 – RO-3306 1 uM
4. Donor 1 – RO-3306 5 uM
5. Donor 1 – RO-3306 10 uM
6. Donor 2 – RO-3306 0 uM, no FN-f
7. Donor 2 – RO-3306 0 uM
8. Donor 2 – RO-3306 1 uM
9. Donor 2 – RO-3306 5 uM
10. Donor 2 – RO-3306 10 uM

Figure 5A – MMP-13

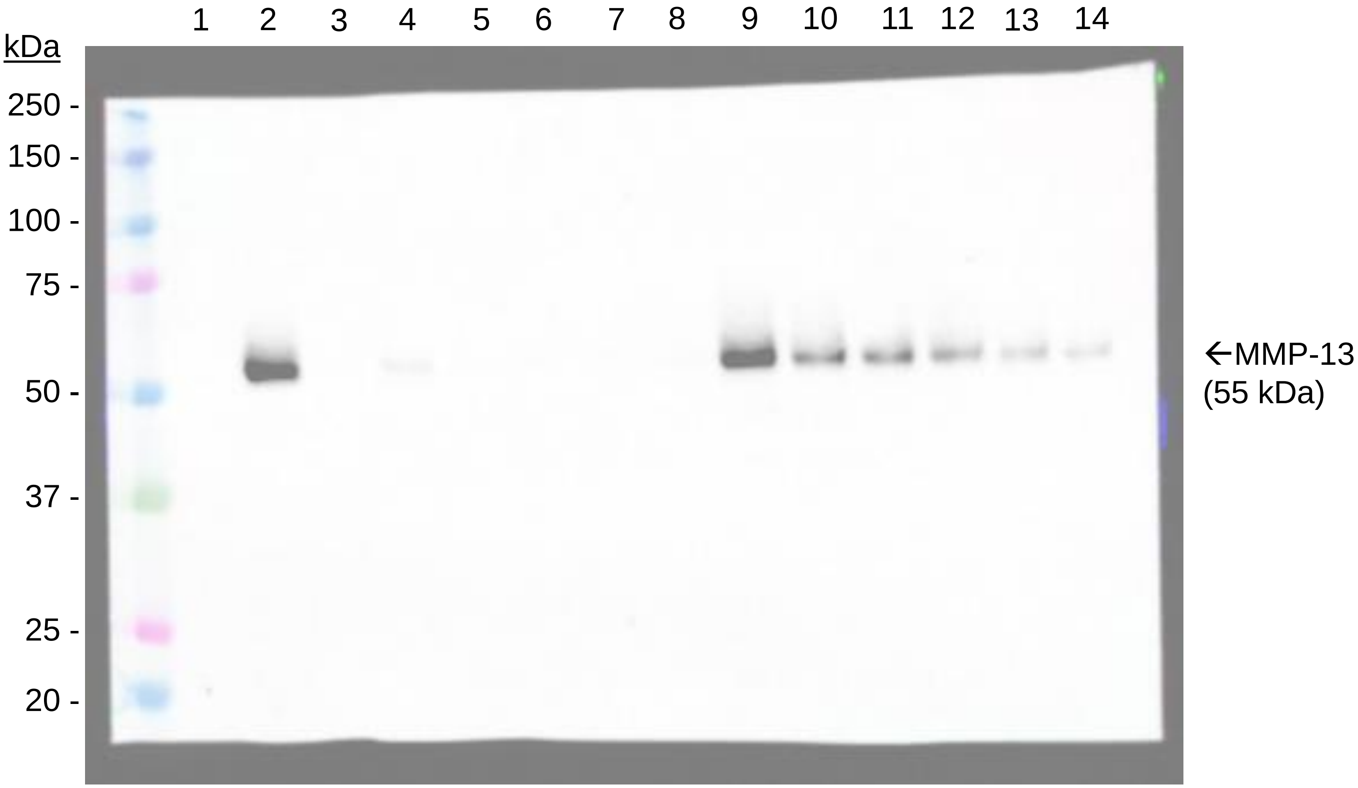

Lanes:
1. No FN-f
2. DMSO
3. Staurosporine 0.14 uM
4. Staurosporine 0.4 uM
5. Staurosporine 1.2 uM
6. Trametinib 0.14 uM
7. Trametinib 0.4 uM
8. Trametinib 1.2 uM
9. Edicotinib 0.14 uM
10. Edicotinib 0.4 uM
11. Edicotinib 1.2 uM
12. GSK-626616 0.14 uM
13. GSK-626616 0.4 uM
14. GSK-626616 1.2 uM

Figure 5A – β-tubulin

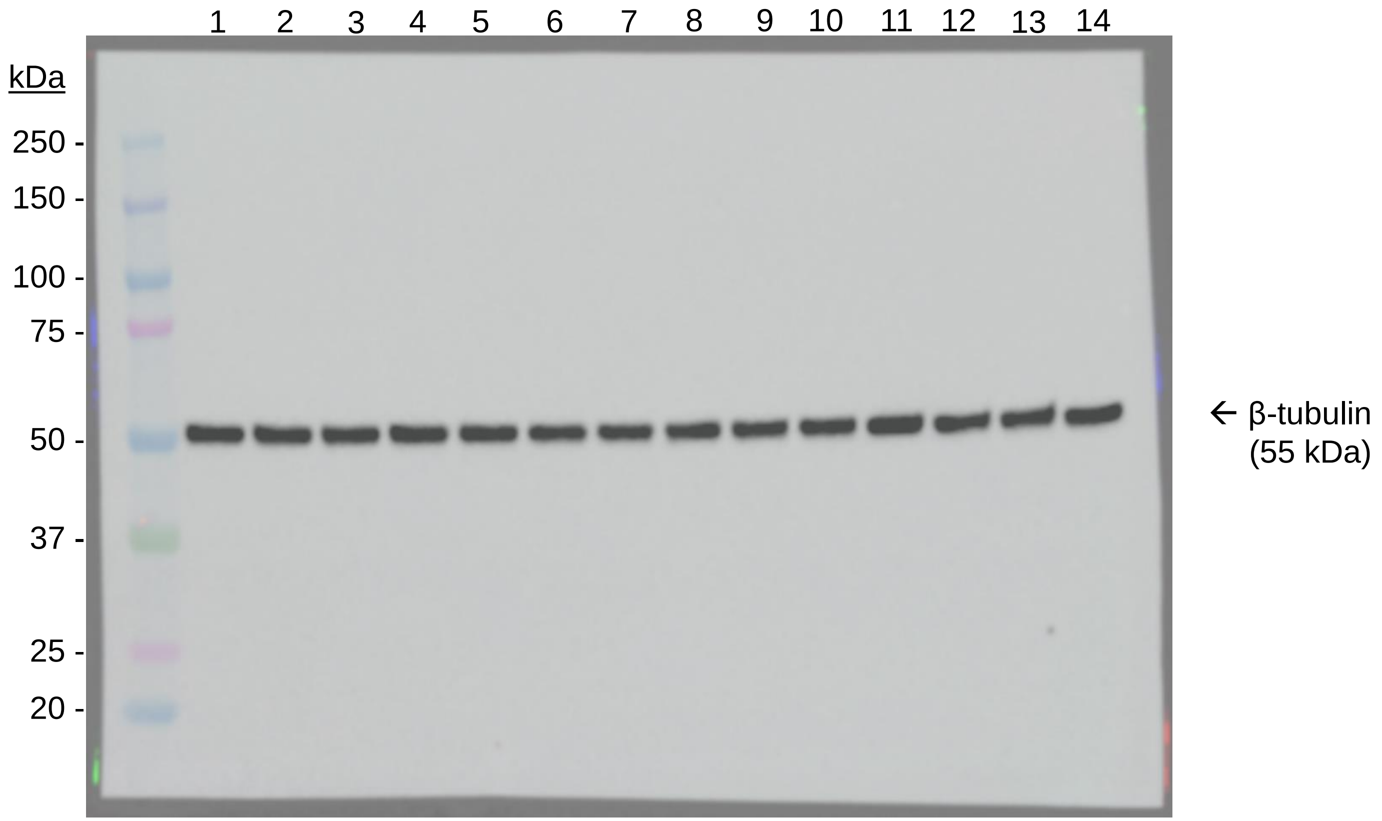

Lanes:

1. No FN-f
2. DMSO
3. Staurosporine 0.14 uM
4. Staurosporine 0.4 uM
5. Staurosporine 1.2 uM
6. Trametinib 0.14 uM
7. Trametinib 0.4 uM
8. Trametinib 1.2 uM
9. Edicotinib 0.14 uM
10. Edicotinib 0.4 uM
11. Edicotinib 1.2 uM
12. GSK-626616 0.14 uM
13. GSK-626616 0.4 uM
14. GSK-626616 1.2 uM

Figure 5C – MMP-13

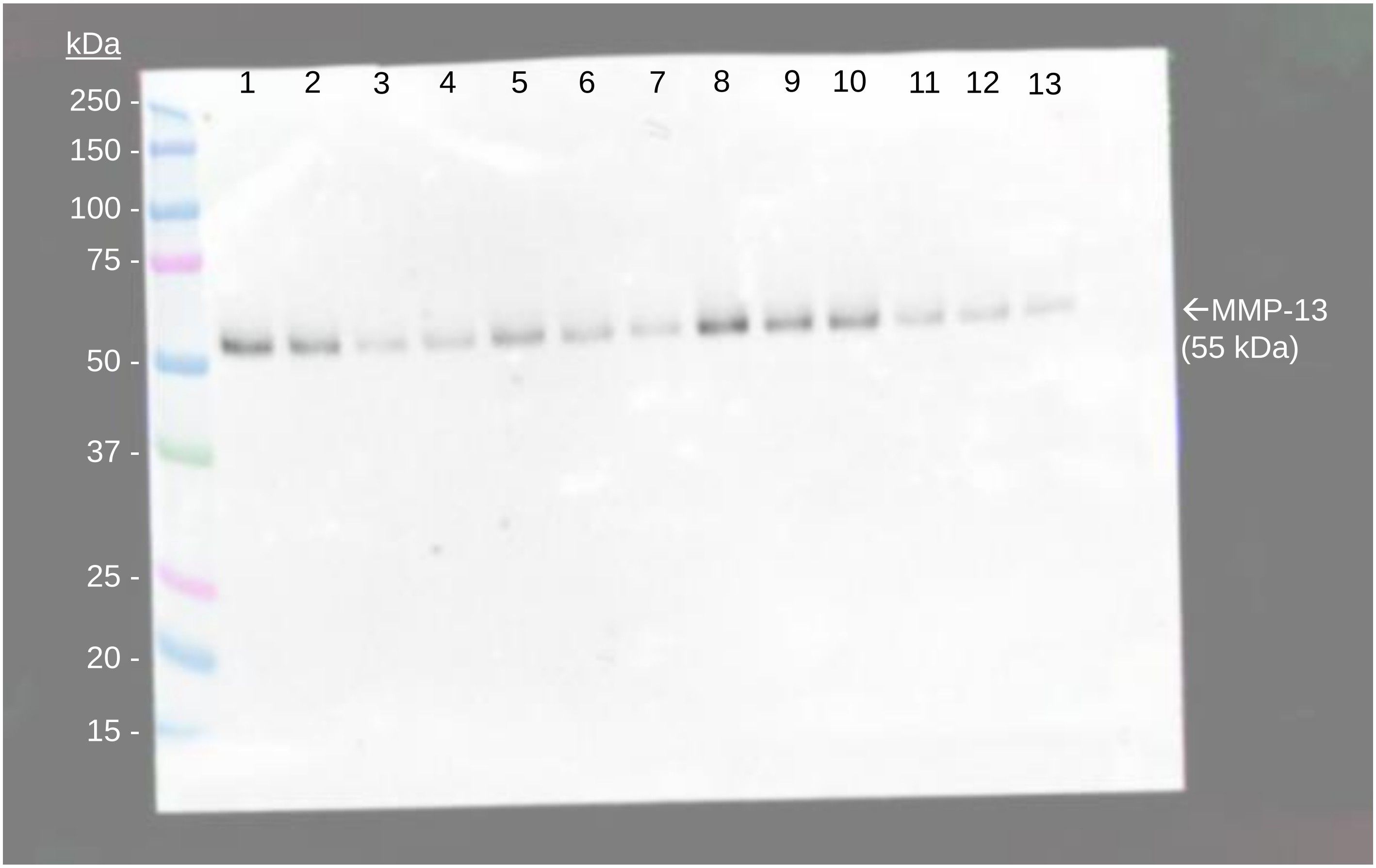

Lanes:
1. DMSO
2. Staurosporine 0.14 uM
3. Staurosporine 0.4 uM
4. Staurosporine 1.2 uM
5. Trametinib 0.14 uM
6. Trametinib 0.4 uM
7. Trametinib 1.2 uM
8. Edicotinib 0.14 uM
9. Edicotinib 0.4 uM
10. Edicotinib 1.2 uM
11. GSK-626616 0.14 uM
12. GSK-626616 0.4 uM
13. GSK-626616 1.2 uM

Figure 5C – β-tubulin

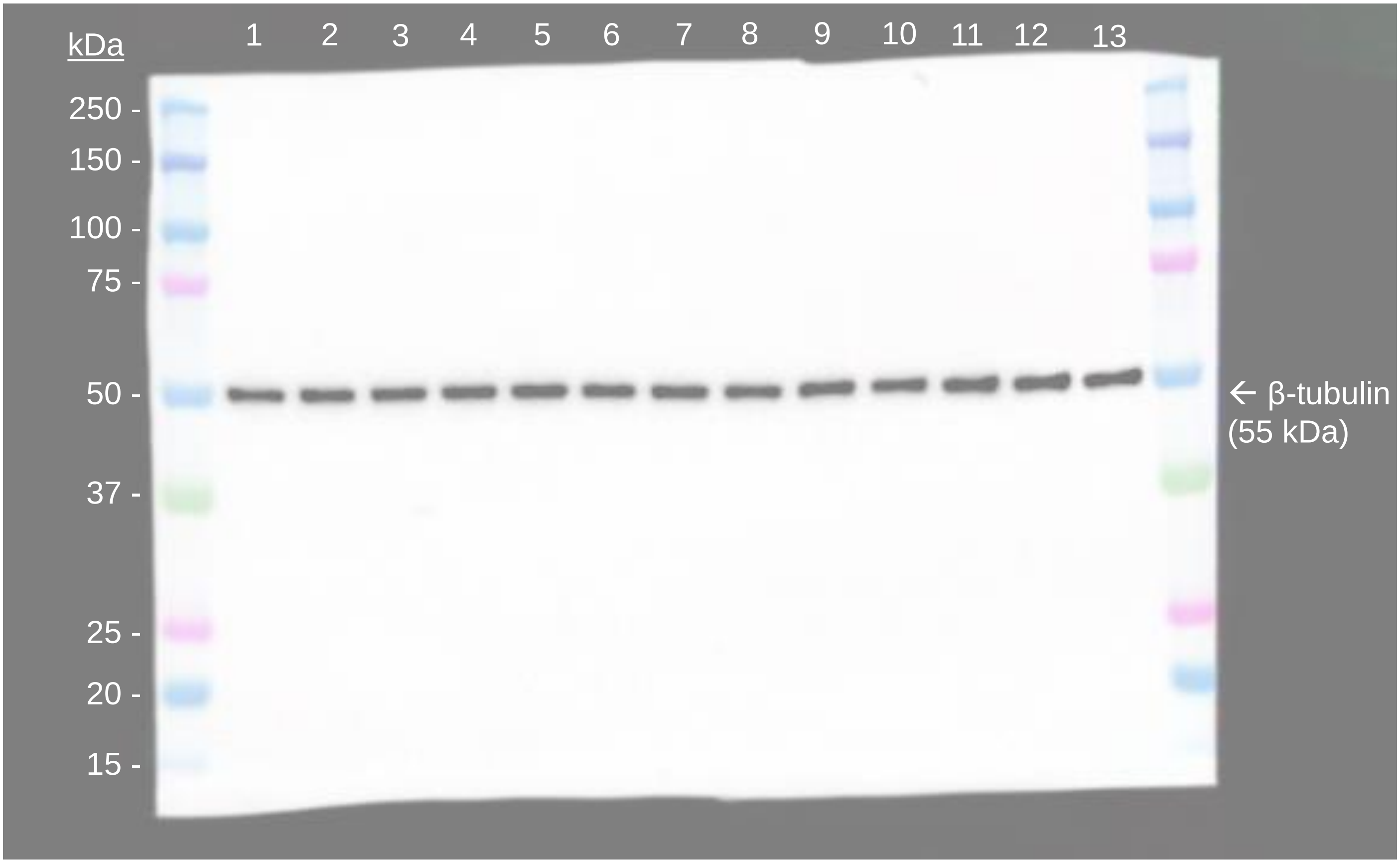

Lanes:

1. DMSO
2. Staurosporine 0.14 uM
3. Staurosporine 0.4 uM
4. Staurosporine 1.2 uM
5. Trametinib 0.14 uM
6. Trametinib 0.4 uM
7. Trametinib 1.2 uM
8. Edicotinib 0.14 uM
9. Edicotinib 0.4 uM
10. Edicotinib 1.2 uM
11. GSK-626616 0.14 uM
12. GSK-626616 0.4 uM
13. GSK-626616 1.2 uM

Figure 6A – IL-6

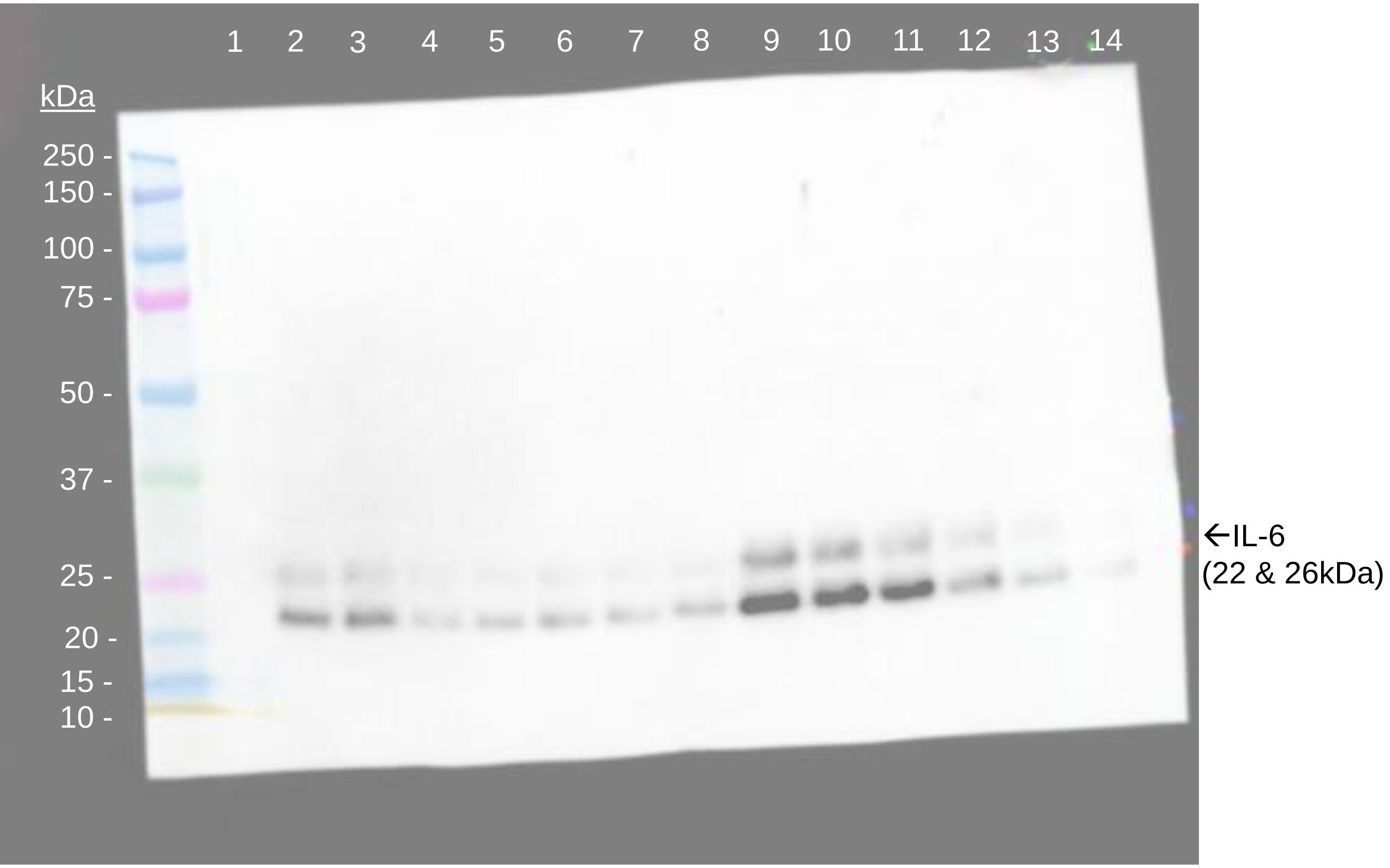

Lanes:

1. No FN-f
2. DMSO
3. Staurosporine 0.14 uM
4. Staurosporine 0.4 uM
5. Staurosporine 1.2 uM
6. Trametinib 0.14 uM
7. Trametinib 0.4 uM
8. Trametinib 1.2 uM
9. Edicotinib 0.14 uM
10. Edicotinib 0.4 uM
11. Edicotinib 1.2 uM
12. GSK-626616 0.14 uM
13. GSK-626616 0.4 uM
14. GSK-626616 1.2 uM

Figure 6A – β-tubulin

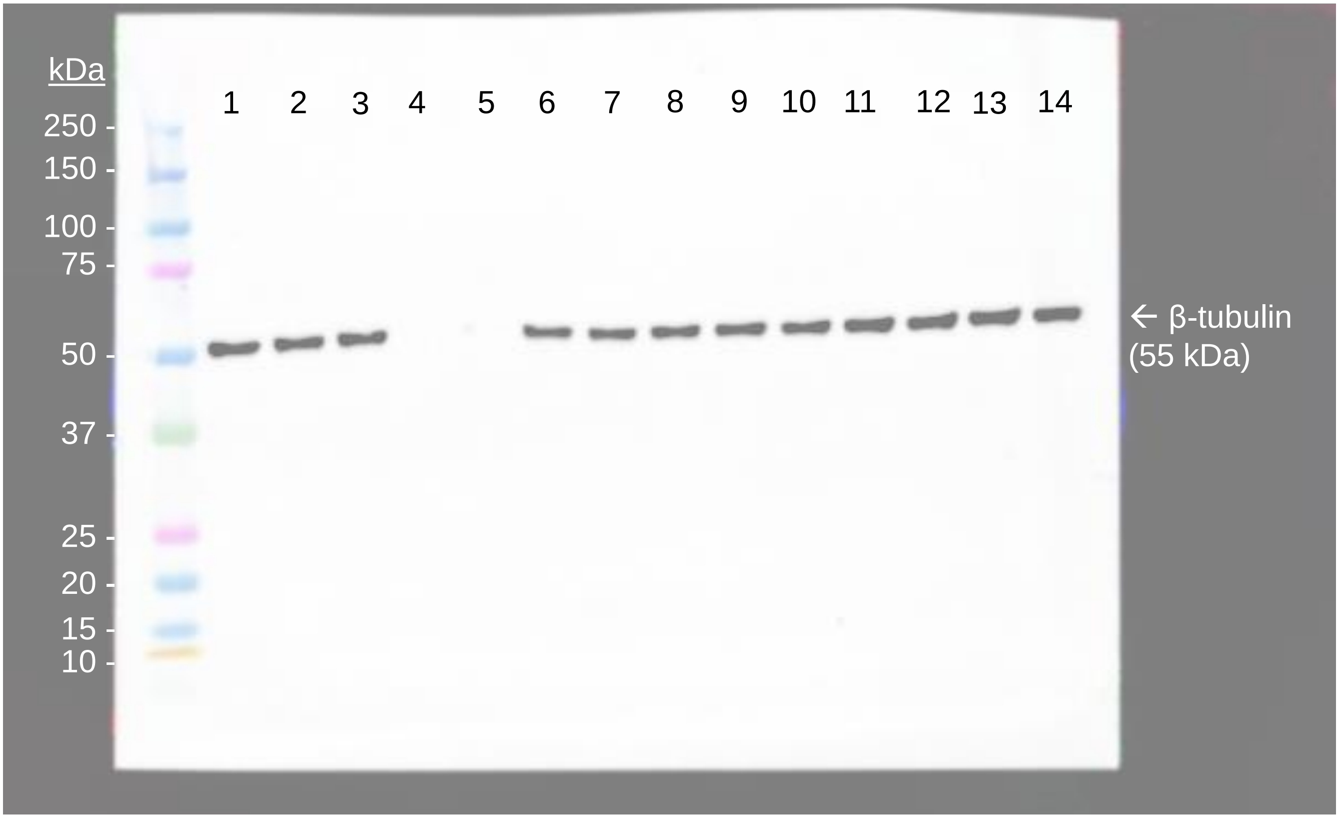

Lanes:

1. No FN-f
2. DMSO
3. Staurosporine 0.14 uM
4. Staurosporine 0.4 uM
5. Staurosporine 1.2 uM
6. Trametinib 0.14 uM
7. Trametinib 0.4 uM
8. Trametinib 1.2 uM
9. Edicotinib 0.14 uM
10. Edicotinib 0.4 uM
11. Edicotinib 1.2 uM
12. GSK-626616 0.14 uM
13. GSK-626616 0.4 uM
14. GSK-626616 1.2 uM
